# Uncovering the Techno-Economic and Environmental Implications of a Multiproduct Biorefinery from Exhausted Olive Pomace

**DOI:** 10.1021/acssuschemeng.4c07901

**Published:** 2025-02-09

**Authors:** Deborah Pérez-Almada, Ángel Galán-Martín, María del Mar Contreras, Juan Miguel Romero-García, Eulogio Castro

**Affiliations:** 1 Department of Chemical, Environmental and Materials Engineering, University of Jaén, Campus Las Lagunillas s/n, Jaén 23071, Spain; 2 Institute of Biorefineries Research (I3B), University of Jaén, Jaén 23071, Spain

**Keywords:** biorefinery, carbon capture storage, exhausted olive pomace, life cycle assessment, techno-economic and environmental assessment

## Abstract

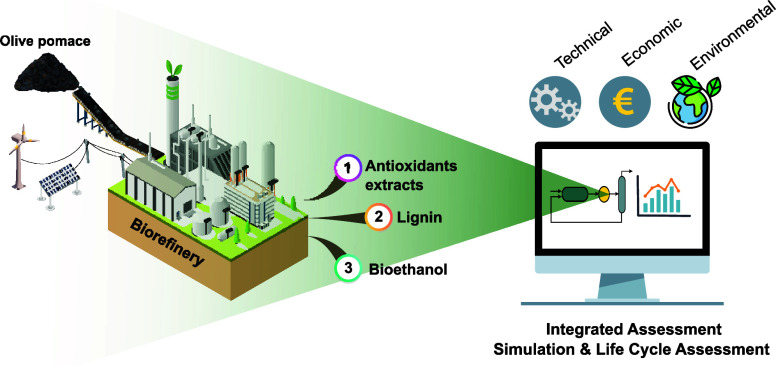

Biorefineries are pivotal in advancing sustainability, yet most studies remain confined to laboratory scales, lacking comprehensive industrial-level analyses. In this work, the laboratory experiments are scaled up to design and assess the techno-economic and environmental implications of a multiproduct biorefinery system producing antioxidant extracts, lignin, and bioethanol from exhausted olive pomace, a residual biomass from olive oil extraction. Using process simulation and life cycle assessment, five scenarios were evaluated, varying in electricity sources (national mix, solar, wind, or olive biomass) and the heat and cooling sources (fossil natural gas or synthetic natural gas from capture CO_2_ and electrolytic hydrogen), with one scenario incorporating a carbon capture and storage (CCS) system. The CCS scenario showed the highest overall costs, 2.5 times higher than the base scenario (27.74 vs 10.99 $/functional unit), primarily due to the additional infrastructure and energy-intensive processes associated with CO_2_ utilization and storage. Despite higher costs, it achieved even a negative carbon footprint (−1.05 kg CO_2_eq per functional unit cradle-to-gate) and reduced impacts on ecosystem quality, resources, and human health. However, specific impacts like human noncarcinogenic and carcinogenic effects (40% and 60%) and ecotoxicity (up 70%) worsened. Notwithstanding economic barriers and environmental challenges, which can be alleviated by selling carbon credits and tailored policies and strategic decisions, these findings underscore the potential of integrating CCS into biorefinery schemes as a promising pathway to enhance environmental sustainability.

## Abstract 2

Scaling up olive pomace biorefineries shows carbon capture boosts sustainability for antioxidants and bioethanol production despite higher costs

## Introduction

Global challenges, such as population growth, resource depletion, and climate change require a paradigm shift in production and consumption patterns.^1^ Biorefineries are positioned to be key drivers in advancing this transition, focusing on processing biowaste resources to drive a sustainable economy built on renewable bioenergy^2,3^ and biobased products.^2^ Governments worldwide are enacting strategies and policies to reduce reliance on fossil feedstocks across all economic sectors and activities. Initiatives like the REPowerEU Plan, the EU “Fit for 55″ package,^4^ the EU Green Deal, the Paris Agreement,^1^ and the United Nations Sustainable Development Goals^1^ underscore a collective commitment to progress in the ecological transition. In this context, deploying biorefineries stands out as an important step in aligning economic activities with sustainability goals, offering opportunities in rural areas, and contributing to a resilient, zero-pollution, and zero-waste bioeconomy.^5,6^

Spain emerges as a potential key hub for biorefinery development within the EU. With its rich agricultural and forestry landscape, Spain holds ample opportunities for biorefinery projects across the country to exploit diverse biomass residues for biobased production processes.^7^ A particularly promising avenue lies within the olive oil sector. As the world’s largest producer of olive oil, Spain generates large amounts of olive-derived byproducts and biomass waste that represent valuable resources for extracting high-value compounds and clean energy.

The olive oil industry encompasses various stages, from olive cultivation to oil production. During the agricultural phase, olive branches and leaves are generated from the pruning process every two years. The olives are harvested, transported, and processed to obtain olive oil, generating olive stones or pits (hard inner seeds found within the olive fruit) and the byproduct olive pomace, which contains the olive pulp, part of the stones and some olive oil. Olive pomace usually undergoes further processing in the extraction industry to extract additional oil with chemicals, generating the exhausted olive pomace (EOP) as residue. Oil extraction generates wastewater with suspended solids, organic matter, and harmful compounds.^8^ These olive-based biomasses, generated both during olive cultivation and oil manufacturing, represent significant resources that can be utilized for various purposes, including bioenergy production or as feedstock to produce value-added products like biofuels or biochemicals.^9^

Among the biomasses generated throughout the olive oil value chains, the EOP stands out as a valuable resource, with Spain alone generating approximately 1.2 million tons per campaign.^10^ The EOP is particularly appealing because it is currently utilized for soil amendment and predominantly for energy or electricity generation; thus, its potential for valorization remains largely untapped. Before resorting to its combustion, there exists an opportunity to unlock additional value from this versatile byproduct. Compounds such as antioxidants and sugar derivatives can be derived from EOP, each with significant market potential and diverse applications. For example, antioxidants can be used in cosmetics and the food and pharmaceutical industries due to their health benefits, while sugars can be converted into bioethanol for transportation.^11^

Several authors have already explored alternative valorization routes for EOP to produce diverse value-added products, finding applications across various sectors. Some studies have explored the valorization of EOP for biofuel production, emphasizing the necessity of efficient pretreatment steps to remove the lignin barrier from the lignocellulosic biomass and release the sugars for effective conversion.^12^ Extracting and delignifying the material through organosolv pretreatment facilitates the extraction of bioactive compounds and enhances enzymatic hydrolysis.^14,15^ These compounds, known for their health-promoting effects due to their ability to neutralize harmful free radicals, highlight the broader potential of EOP beyond its traditional uses.

In addition to the antioxidant extracts, the fermentable sugars in the EOP, once released through pretreatment (e.g., hydrolysis), can be converted to platform chemicals or biofuels, offering a renewable alternative to traditional fossil fuels. This pathway is particularly interesting from a macroeconomic perspective as it will play a role in greenhouse gas (GHG) mitigation and energy independence.^18^ Bioethanol production from EOP has been investigated through various pretreatment and fermentation methods. Dilute acid hydrolysis followed by enzymatic treatment effectively releases fermentable sugars from EOP, though some residue may remain resistant to cellulase hydrolysis.^13^ Detoxification steps, such as overliming or activated charcoal treatment, are often required to remove inhibitory compounds before fermenting hemicellulosic sugars.^14^ A cascading approach has also been proposed to sequentially extract antioxidants, lignin, and sugars, with the latter being suitable for conversion into bioethanol.^15^

Additionally, lignin, a high molecular weight aromatic polymer also abundant in EOP, finds applications in various industries, as a precursor for biofuels, materials, and chemicals. This multifaceted utilization of EOP underscores its potential to contribute significantly to sustainable industrial practices.

All previous research predominantly centered around experimental studies, delving into the fundamental aspects of EOP valorization at the laboratory scale. While these studies have substantially contributed to understanding EOP management and utilization, there remains a critical gap in the literature regarding the practical implications of implementing such valorization routes on an industrial scale.

Among the tools available, integrating process simulation tools with life cycle assessment (LCA) methodologies has emerged as a robust approach for comprehensively understanding the technical, economic, and environmental implications of biorefining systems on a large scale.^16^ Process simulations provide a framework for approximating the performance of biorefinery processes at an industrial scale based on laboratory data, facilitating the identification of scaling challenges. However, such simulations should be complemented by rigorous experimental and engineering scaling-up procedures to ensure robustness and reliability. Subsequently, the data generated from these simulations can be utilized to analyze both the economic feasibility and the environmental footprint of the processes. For the latter, LCA is a widely used methodology that offers insights into the environmental footprint of biorefinery systems across their entire life cycle and considers several impact categories.^17^ The combined approach of Environmental and Techno-Economic Assessment (ETEA) has been applied to explore the valorization of olive byproducts^18^ such as bioethanol production from olive tree pruning (OTP).^19−21^ However, these previous studies have predominately centered on olive biomasses other than EOP, with some narrowly focusing on the technical and economic aspects while neglecting the environmental (and social) dimension, which risks overlooking potential undesired side effects.

To address these gaps, this study conducts a comprehensive simulation exercise to model an industrial-scale operation, starting from laboratory studies on EOP valorization^15^ and extending to an in-depth examination of the technical feasibility, economic viability, and broad environmental implications of a multiproduct EOP-based biorefinery.^22^ An integrated ETEA approach was adopted, providing a robust framework for evaluating various biorefinery schemes focusing on the sustainable valorization of EOP as biomass waste. This approach provides valuable insights into integrating diverse renewable energy sources within the biorefinery and evaluating pathways involving carbon capture, utilization, and storage technologies.

By examining different biorefining scenarios, this study offers a comprehensive understanding of how different strategies can be optimized for sustainability and economic performance in the biorefinery sector. By doing so, the aim is to offer valuable insights and practical guidelines for the sustainable management and utilization of EOP, ultimately contributing to developing a resilient, zero-waste, and low-carbon bioeconomy.

## Experimental Section

### Process Description and Simulation

The simulation design of the multiproduct biorefinery plant was carried out using Aspen Plus v11.0.^23^ The plant has a capacity of 100,000 tons/year of EOP (6.5% moisture), chosen based on EOP availability in Andalusia, southern Spain. The region produces over one million tons of EOP annually, with more than half used for electricity generation. Two extraction facilities in Baena and Lucena alone generate approximately 150,000 tons/year.^10^ The selected capacity, representing about 8% of regional production, ensures a reliable feedstock supply while optimizing operational efficiency and maintaining manageable transportation costs.

The final biobased products of the plant include antioxidant extracts, lignin, and bioethanol. Experimental data and results from a previous study^15^ were used as a starting point and the simulation dry matter composition belonged to solid was essentially composed of lignin (38%), xylan (16%), cellulose (16%), ash (11%), and protein (11%). The nonrandom two-liquid property model was used in the simulations and components in the Aspen simulation to model the liquid. At the same time, the Hayden-O’Conell equation-of-state is ideal for accurately predicting complex nonideal systems and phase behavior (usage in high-pressure processes). All reactors were conversion reactors (RStoic), modeled based on stoichiometric relationships found in the Supporting Information Material (Tables S2–S5). The simulation process is continuous with a total of 7620 h/yr, and the use of pseudocomponents where applicable, along with their defined properties. All component definitions can be found in Supporting Information Material (Table S1). Further details regarding the simulation models, including stream compositions and process flow diagram, and mass and energy balances, are reported in section 1 of Supporting Information Material (Tables S1–S6).

**Figure 1:**
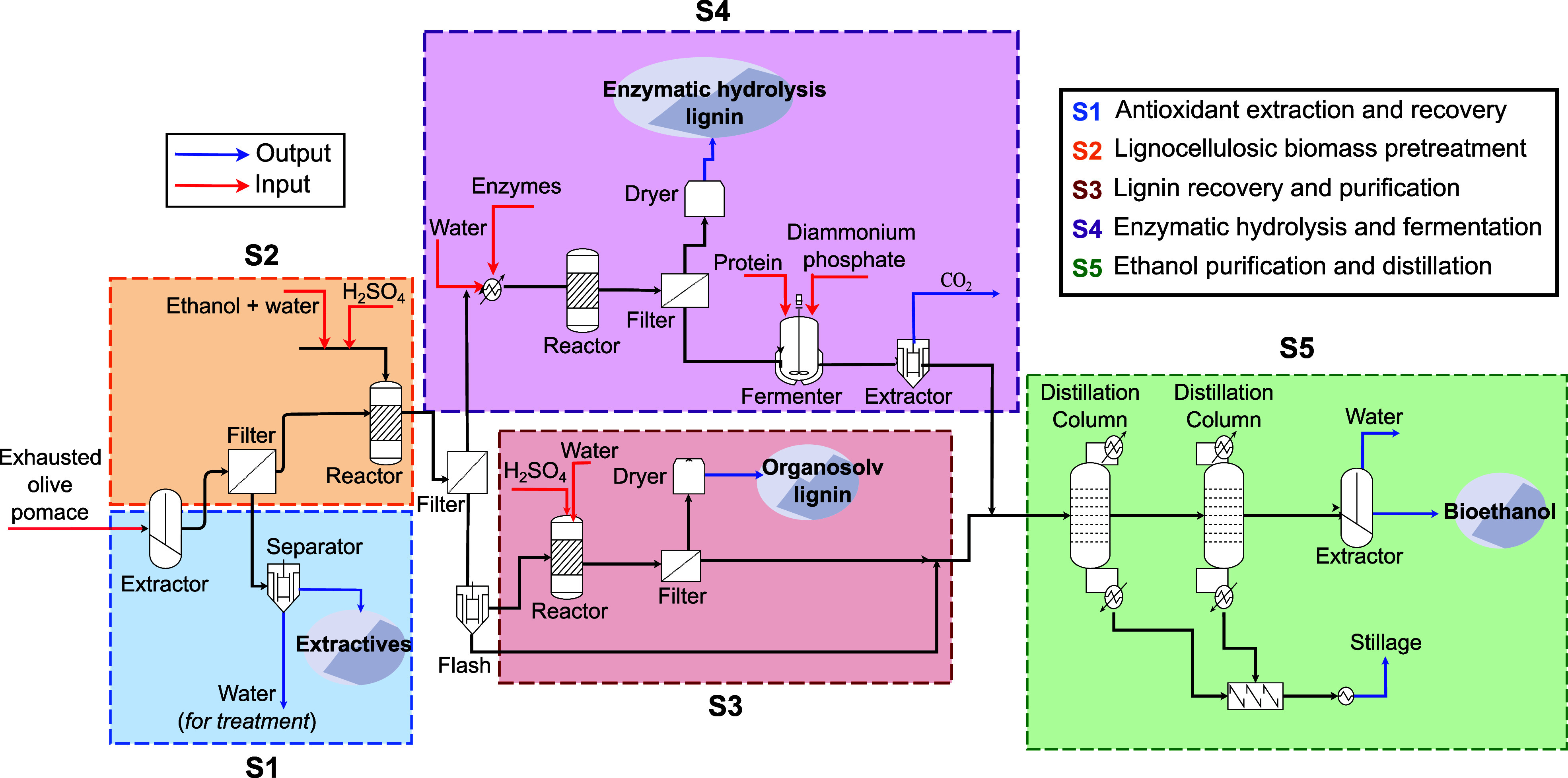
Overview of the process flowsheet of the multiproduct biorefinery converting exhausted olive pomace into antioxidant extracts, lignin, and bioethanol.

A schematic representation of the multiproduct biorefinery process is presented in Figure 1, which is divided into the following subsystems: (S1) Antioxidant extraction and recovery, (S2) Lignocellulosic biomass pretreatment, (S3) Lignin recovery and purification, (S4) Enzymatic Hydrolysis and fermentation, and (S5) Ethanol purification and distillation.

In S1, according to the laboratory experiments^1,^ the EOP was subjected to an aqueous extraction at 10% mass concentration (w/v) of solids and introduced in a reactor at 85 °C. Then, the wet material was filtered and separated into an aqueous and solid fraction. The aqueous extraction allowed a recovery efficiency of more than 75% of the initial EOP mass, which is mainly composed of antioxidants, nonstructural sugars, mannitol, and phenol.

The extract is rich in antioxidants (yield of 5795.49 kg/h) and contains around 10% hydroxytyrosol, an important phenolic compound due to its chemical properties. Additionally, the extract component was rich in bioactive compounds such as tyrosol and mannitol with around 10%.^15^ After the antioxidant extracts were recovered, wastewater was obtained at the bottom of the separator block (flash block) and sent to a wastewater system. The solid recovered from the water extraction step (around 59% of the original biomass) enters S2, where it is pretreated by carrying organosolv pretreatment using sulfuric acid at 1% (w/v) and 50% ethanol. The solid fraction was introduced in a reactor with a target temperature of 130 °C. The pretreated EOP contains antioxidant extracts, lignin, polymers of glucose, and xylose. Next, the stream was cooled to around 30 °C, and the obtained slurry was vacuum filtered and separated in a pretreatment liquor (PL) and pretreated solid (PS). For the lignin recovery (S3), the solubilized lignin was precipitated from the PL by adding two volumes of acidified water with sulfuric acid. This PL was evaporated up to 50% of the volume by using separator equipment. Consequently, the resulting fraction was filtered and dried at 50 °C and the obtained product was called organosolv lignin (OL) with a yield of 495.83 kg/h.

In S4, another method for obtaining lignin involved recovering the solid fraction from the enzymatic hydrolysis-fermentation stage. The main aim of this S4 subsystem is converting pretreatment lignocellulosic biomass into fermentable sugars by breaking down complex organic compounds through the addition of cellulases (enzymes). In essence, the PS was subjected to enzymatic hydrolysis by using an enzyme and performed at 50 °C. The reaction products were vacuum filtered and separated in hydrolysate and solid fraction. Consequently, the solid fraction was named enzymatic hydrolysis lignin (EHL) to be composed mainly of lignin. The EHL (yield of 1334.02 kg/h). For the bioethanol conversion, the hydrolysate fraction was sent to a fermentation process reactor at 30 °C. After finishing the fermentation reactions, the final fermented product is ready to be sent to the distillation columns for bioethanol production together with the evaporated and bottom fraction from the S3. The ethanol purification stage (S5) consists of two distillation columns during the separation/purification of the ethanol phase to reach a purity level near the azeotropic point. In the first distillation column, the ethanol is concentrated to about 51% (w/w). The top ethanol column goes to the rectification column (second column) to reach a value near its azeotropic point (95.3%). Then, the resulting bioethanol (yield of 246.38 kg/h) was purified to about 99% using molecular sieves, which can be used as biofuel for transportation. The bottoms of the columns are collected as stillage (mainly composed of water and lignin) and then filtered. The filtered stillage can be further processed to extract added value, such as through anaerobic digestion to produce biogas, which can subsequently be used for power generation. Additionally, treated water can be recovered and reused.

### Scenario Definition

**Figure 2:**
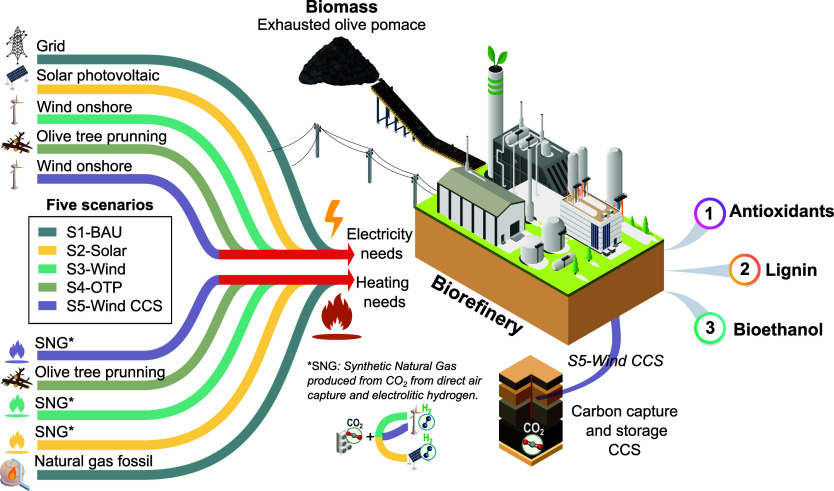
Graphic overview of the multibiorefinery system scenarios considered in this study. The scenarios are color-coded (as shown in the legend on the left) and differ based on their electricity sources and heat/cooling utilities.

Considering the biorefinery plant described before, five scenarios are defined, as depicted in Figure 2. The goal is to compare the economic and environmental impacts of the scenarios and identify opportunities for improvement. The scenarios vary in the electricity source powering the processes (Spanish grid, solar photovoltaic, wind power, and bioelectricity from olive tree pruning biomass) and heat/cooling source (fossil natural gas or synthetic natural gas generated from Sabatier reaction of captured CO_2_ and electrolytic hydrogen) while one scenario integrates a carbon capture and storage (CCS) system to capture the CO_2_ emissions both from the synthetic natural gas (SNG) combustion and the fermentation unit. In the base case scenario, labeled as the business-as-usual scenario (S1-BAU), the power is generated from grid electricity while the heat needs are covered by steam from fossil natural gas. Two alternative scenarios utilize SNG for heating/cooling needs, produced following a carbon capture and utilization (CCU) pathway. The SNG is produced through the Sabatier reaction of CO_2_ from direct air capture (DAC) and electrolytic H_2_ powered by solar photovoltaic or wind (scenarios S2-Solar and S3-Wind, respectively). DAC plants are facilities that use chemical solvents and sorbents to extract CO_2_ directly from the atmosphere. For electricity needs, both scenarios (S2–S3) utilize these renewable technologies, with the grid serving as backup due to their intermittent nature, considering average capacity factors (20% for solar and 30% for wind).^24^ The fourth scenario considers the cogeneration of heat and power from olive tree pruning (S4-OTP). In olive tree cultivation, OTP is generated biennially during pruning operations after fruit harvesting. Its availability depends on factors such as tree age, plantation density, soil and climate conditions, and irrigation practices. Note that, within the olive oil production value chain, OTP residues represent an abundant and underutilized source of wood fuel, holding immense potential to be exploited.^25^ Finally, in the fifth scenario (S5-Wind CCS), akin to S3-Wind, a CCS system based on monoethanolamine solvent with a 90% capture efficiency is integrated into the biorefinery plant. It captures both the CO_2_ emissions from combusting the SNG and the biogenic CO_2_ from the fermentation unit, which is then compressed and transported via pipeline for injection into geological storage sites. This scenario S5-Wind CCS represents a promising long-term carbon removal alternative due to its dual approach of removing CO_2_ emissions from the atmosphere via DAC technology and biogenic sources, thus contributing to climate change carbon reduction goals.

### Economic Analysis

The economic viability of the EOP-based multibiorefinery was estimated using the methodology, correlations, and factors described in literature references^20,26,27^ and Turton et al. (Chapters 7–10).^28^ The process simulation in Aspen Plus provides the equipment size, material, and energy flows that are essential to estimate the revenues, capital (CAPEX), and operational (OPEX) expenditures. The economic indicators determined were Net Present Value (NPV), Payback Period (PP), and Return of Investment (ROI). We assume a lifespan of 25 years with 7920 h per year of operation and a 5% interest rate. Revenue streams considered in this assessment included the sale of the main biobased products bioethanol, lignin, and extractives.

The cost parameters for the utilities, products, and material prices are provided in Supporting Information Material section 4 in Tables S10–S15. The CAPEX parameters were calculated by using the bare module cost of each equipment *k (Cbm*_*k*_*)* as shown in eq 1. Additionally, in eq 1 the Chemical Engineering Plant Cost Index (CEPCI), was used in the formula to update the costs from 2018 to 2022 to calculate the CAPEX. Consequently, the interest rate (IR) and year lifetime (years) were needed to calculate the capital annualization factor (AF) in eq 2. Finally, the total annualized cost (TAC) was employed by using the sum between the OPEX and the annualized capital cost AF, and CAPEX as shown in eq 3.
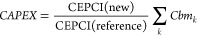
1
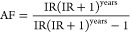
2
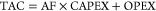
3

Regarding the OPEX of the multibiorefinery plant, the global average costs for the utilities, raw materials, electricity and product prices were used. A sensitivity analysis was conducted to account for variability and improve the robustness of the assessment. In the eq 4, the *MB*^*F*^ and *MB*^*W*^ are the total costs of the feedstock materials and water fed in the plant, respectively; *MB*^*H*^ is the cost of heating; *MB*^*E*^ and *MB*^*C*^ are the total costs of electricity and cooling, respectively. The OPEX data for each scenario was collected from literature data and shown in the Supporting Information Material in section 4.2 (Tables S13–S15).

The equation used is eq 4 shown below:
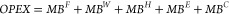
4

A complete and detailed explanation of the CAPEX and OPEX calculations and parameters can be found in sections 4.1 and 4.2 in the Supporting Information Material.

A sensitivity analysis of the economic results was performed across the five biorefinery scenarios to evaluate the impact of variations in key parameters on economic outcomes. The central scenario represents average values, while the pessimistic and optimiztic scenarios account for potential negative and positive deviations. These variations in cost parameters are defined based on historical data, regional variations, and market fluctuations, as described in Section 4.2 of the Supporting Information Material. Exploring these scenarios provides a clearer understanding of the sensitivity of economic indicators to changes in key factors, including material costs, energy prices, and operational efficiencies. This sensitivity analysis helps to identify the most critical variables influencing the financial viability of the biorefinery and provides valuable insights for risk management and strategic planning.

Additionally, a breakeven point analysis and pricing strategy for carbon removal credits are performed. The breakeven point analysis allows us to determine the minimum selling price required for the biobased products to be economically viable. This analysis is crucial as it reveals the price at which the revenue from selling the biobased products from the multibiorefinery scenarios surpasses those produced from fossil fuel resources, specifically natural gas as used in the S1-BAU scenario. The analysis involved incrementally adjusting both the feedstock and selling prices across all five scenarios (S1–S5) until each scenario achieved an equilibrium NPV, thus revealing the price competitiveness required for market parity.

Finally, a complementary analysis was conducted to support the S5-Wind CCS scenario, which involved evaluating the impact of premium pricing strategies and the potential revenue from carbon credits. The incorporation of carbon credit sales for each ton of CO_2_ sequestered was analyzed to evaluate how such financial mechanisms could enhance the economic attractiveness of the S5-Wind CCS scenario. This analysis aimed to explore how leveraging carbon credits and premium pricing could offset higher production costs and improve the financial feasibility of integrating CCS technologies into the biorefinery.^22^

### Environmental Assessment

LCA is a systematic and comprehensive method that allows examining the environmental impacts associated with the biorefinery system throughout its entire life cycle.^29^ The standardized methodological framework procedures are provided by ISO 14040^30^ and ISO 14044^31^ and were followed to assess and compare the biorefinery scenarios described previously.

The first stage is the definition of goal and scope. This study aims to examine and compare the environmental performance of EOP multibiorefinery in different scenarios. The functional unit (FU) selected for this analysis is the production of 1 kg of bioethanol, 0.49 kg of antioxidant extracts, and 0.98 kg of lignin. This multifunctional FU was chosen to simplify the analysis by avoiding complexities associated with economic allocation methods or system expansion techniques. The primary focus is not on determining the best management of EOP waste but on identifying hotspots within a system and providing an unbiased basis for comparing the five standalone scenarios.^32^

The scope adopted was from cradle-to-gate, namely from the olive production and EOP acquisition and processing to the delivery of biobased products at the biorefinery gate.

The second phase is the life cycle inventory (LCI), which consists of compiling the inputs and output flows connected to the biorefinery system (feedstock, products energy, emissions, waste, etc.) across its full life cycle.^29^ Data for the foreground system (mass and energy balances entering and leaving the biorefinery plant) were derived from the simulation model built in Aspen Plus based on the experimental results. The results of the simulation are described in section 2 of the Supporting material. Data from the background system (e.g., wind and photovoltaic system power sources) were primarily obtained from data sets available in Ecoinvent v3.9.1^33^ except for the EOP raw material, OTP feedstock, and the SNG.

The LCI of the EOP was developed by modeling the complete olive oil production industry based on a previous study^9^ and applying economic allocation to the EOP. For the OTP feedstock, we performed economic allocation using the Olive fruit production – ES data set from Ecoinvent v3.9.1,^33^ allocating inputs and outputs between olive fruit and prunings. Chipping, mulching, and transportation (50 km by lorry) were also considered. Impacts from OTP storage at the biorefinery were assumed to be negligible. The direct combustion emissions for the S4-OTP were sourced from another article.^25^ The inventory tables regarding the LCA of olive oil production, including economic allocation parameters, can be found in section 5 in the Supporting Information Material (Tables S16–S17). For the SNG generated from the Sabatier reaction of captured CO_2_^34^ and electrolytic hydrogen,^35^ the LCA inventory and the water electrolysis inventory were obtained from various literature references.^36−38^ In particular, the LCI for the CO_2_ from DAC is sourced from Keith et al. (2018)^39^ (Supporting Table S24), while the postcombustion CCS infrastructure was sourced from the study by Volkart et al. (2013) and Terlouw et al. (2021), which include the transport, pipeline, and drilling processes.^40,41^ Regarding the CCS system, a 90% capture efficiency was assumed.

The third phase is the life cycle impact assessment (LCIA), which translates the inventory flows into quantifiable and understandable environmental impacts, aiding in the interpretation of the overall environmental footprint of the biorefinery. The end point and midpoint categories from ReCiPe 2016 methodology were employed (v1.03).^42^

Using the SimaPro software^43^ (version 9.5.0.0), the LCI was built and calculated the environmental impacts considering the 18 midpoints and three end point environmental impact categories related to human health, ecosystem quality, and resource depletion areas. The end point results and their underlying midpoint category contributions are presented in the main manuscript (Figure 6), offering a broader perspective on environmental impacts. Detailed midpoint results, capturing specific environmental effects, are provided in the Supporting Information Material (Table S39). This comprehensive approach ensures transparency and allows readers to assess the overall environmental performance and the specific contributions of individual impact categories, supporting a more informed interpretation.^44^

The ReCiPe methodology sets the characterization factor for biogenic CO_2_ to zero, meaning biomass-derived products do not receive credit for CO_2_ removed from the atmosphere during photosynthesis. To address this issue, biogenic CO_2_ uptake in biomass-derived products was separately and manually accounted for, as required by ISO 14067.^45^ Note that a cradle-to-gate approach was adopted in this study, which excludes the end-use phase. However, the CO_2_ embodied in OTP-derived products such as bioethanol will likely be released back into the atmosphere during the end-use phase, depending on the disposal or utilization pathway. To comply with standards, the results for the carbon footprint will be provided both with and without accounting for biogenic CO_2_. Specifically, the CO_2_ embodied in the EOP is considered a negative entry of biogenic CO_2_ in the system, estimated based on a moisture content^15^ of 6.5% and carbon content on a dry basis of 45.41%.^46^ Additionally, for the CO_2_ captured from DAC and used as feedstock for SNG production in the S5-Wind CCS scenario, the permanently sequestered CO_2_ is modeled as a negative entry of fossil CO_2_, contributing as a carbon-negative component in the overall emissions accounting.

The fourth and final phase of the LCA study is the interpretation. A sensitivity analysis was performed to assess the robustness of the carbon footprint results and conclusions, considering the uncertainty in the LCI data sourced from Ecoinvent v3.9.1.^33^ Uncertainties related to the foreground system, such as mass and energy balances derived from the process simulation, were excluded from this analysis due to the need for additional assumptions and the potential for increased computational complexity. The Monte Carlo sampling method in Simapro v9.5.0.0 was employed, running 1,000 scenarios based on the probability functions modeling the LCI elementary flows, as defined in Ecoinvent v3.9.1.^33^ The uncertainty results were displayed for each scenario using error bars spanning the 25th and 75th percentiles (i.e., interquartile range representing 50% of the central results).

## Results and Discussion

### Economic Results

**Figure 3:**
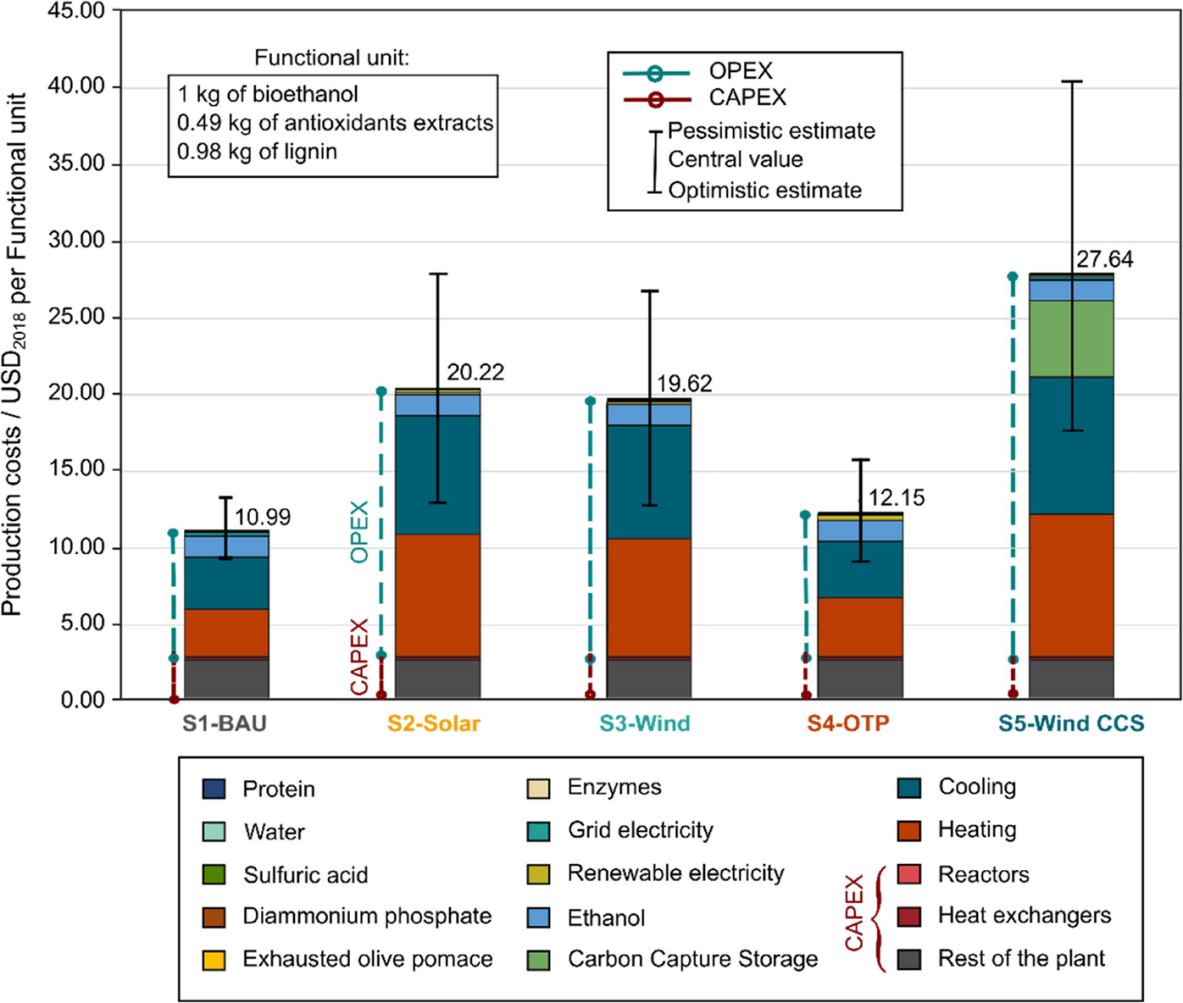
Cost breakdown of exhausted olive pomace multibiorefinery production for the different scenarios. The CAPEX of columns, vessels, and pumps comprise the “rest of the plant” category. Error bars spans the pessimistic and optimistic cost estimates.

The total cost and its detailed breakdown per FU (comprising 1 kg of bioethanol, 0.49 kg of antioxidant extracts, and 0.98 kg of lignin) are first evaluated across the five biorefinery scenarios (Figure 3). Considering the current average prices for raw materials and utilities (central values over bars in Figure 3), S1-BAU shows the lowest overall costs at 10.99 $/FU. This is primarily due to its reliance on cheaper heating and cooling duties derived from imported natural gas, yet it is subject to market forces and fluctuating prices.^47,48^ Even under the pessimistic scenario, the S1-BAU still stands up as the most economically appealing scenario due to the high upfront costs associated with building the infrastructure of the other scenarios.

However, relying solely on imported fossil fuels is risky due to the high uncertainty in prices and dependency on abroad, unlike the alternative scenarios that leverage domestic resources such as solar, wind, or biomass. Noteworthy is the S4-OTP scenario, emerging as a compelling alternative in terms of cost efficiency, with total costs only 10.55% higher than the baseline, amounting to 12.15 $/FU. Still, under the most optimiztic cost estimates, the S4-OTP scenario may achieve lower costs compared to S1-BAU scenario (Figure 3). This potential cost advantage is attributed to utilizing locally sourced OTP for the heating requirements, which reduces transportation and feedstock costs (ranging from 12.33 to 9.05 $/FU in the S4-OTP scenario).

Given the potential location for the biorefinery in Andalusia, where olive grooves dominate the landscape, the required OTP feedstock for the S4-OTP scenario would be available nearby. The S4-OPT demands approximately 259.35 kg of OTP per hour, totaling 1,976.3 tons/year over 7,620 operating hours. With an average OTP yield of 2.7 tons/ha/year, an olive groove spanning 730 ha, equivalent to a 1.5 km radius, would be sufficient. This proximity minimizes transportation costs, enhancing both economic viability and competitiveness.

Moreover, the high efficiency and advanced technology for combustion enhance economic viability and lower costs.^27^ Furthermore, S4-OTP strategically relies on domestic biomass resource residues, thereby mitigating risks associated with external market fluctuations and positioning this alternative as an attractive option for sustained economic stability.

Scenarios relying on renewables and SNG would substantially escalate the costs. The S2-Solar, for instance, has an almost 2-fold increase in cost (20.22 $/FU), while the scenario S3-Wind (19.62 $/FU) compared to the baseline S1-BAU. The breakdown of costs by feedstocks and utilities evidence that the higher costs arise from the expensive heating and cooling needs covered with SNG relying on captured CO_2_ from DAC and electrolytic hydrogen. On one hand, capturing CO_2_ from the atmosphere where concentrations are low using DAC technology requires a substantial energy input, making this CO_2_ raw material costly (from 134 to 342 $/t CO_2_).^49^ On the other hand, the production of green hydrogen is similarly expensive, particularly at 5.66–7.55 $/kg and 4.28–6.86 $/kg when produced from solar and wind power, respectively (S2-Solar and S3-Wind scenarios). Overall, the costs for heating utilizing SNG are 63% and 65% higher than those using fossil natural gas (for the S2-Solar and S3-Wind scenarios, respectively).

The S5-Wind CCS scenario emerges as the most expensive option, with costs tripling to reach 27.74 $/FU compared to the baseline S1-BAU. This increase is primarily attributed to the higher SNG costs as well as the extra costs associated with the infrastructure required for the CCS system (capturing both the CO_2_ from combusting the SNG and the biogenic CO_2_ from the fermentation unit. Notably, beyond the economic barriers and other regulatory and permitting obstacles, the successful implementation of this scenario presents even greater challenges, requiring the strategic identification of sites that offer proximity to the biomass feedstock and suitable geological formations for CO_2_ storage, ensuring economic viability.

Hence, Spain’s diverse geological context, which includes abundant carbonate and mafic-ultramafic rocks, potentially provides a high capacity for secure geological CO_2_ storage (i.e., 14.779 Mt CO_2_ together with Portugal).^50,51^

Concerning the financial metrics, OPEX outweighs the CAPEX in all scenarios, accounting for 75% (S1-BAU), 87% (S2-Solar), 88% (S3-Wind),79% (S4-OTP) and over 90% (S5-Wind CCS).

The CAPEX distribution comprises between 25 to 13% depending on the scenario. The “Rest of the plant” category, which includes the pumps, dryers, filters, towers, and other equipment, accounts for over 90% of the total CAPEX costs, followed by the heat exchangers (4%), while the remaining goes to the reactors.

In terms of the NPV (considering central cost data), only the S1-BAU and S4-OTP scenarios are viable, with $54.25 and $46.04 million, respectively. In contrast, the other alternative scenarios exhibit negative NPV of -$20.97 million for S2-Solar, -$16.12 million for S3-Wind, and -$111.64 million for S5-Wind CCS. This negative NPV affects other financial metrics, such as the PP and the ROI. For example, the PP for both S1-BAU and S4-OTP is approximately 9 years with ROI of around 11%. Conversely, the S2-Solar, S3-Wind, and S5-Wind CCS scenarios present PP ranging from 12 to 13 years and ROIs between 9% and 10%. While these scenarios may provide environmental advantages and reduce the reliance on fossil fuels, their extended payback periods and relatively modest returns make them less attractive from an investment perspective. Notably, when considering the most optimiztic parameters for the renewable energy and SNG costs, all scenarios achieve positive NPV values except the S5-Wind CCS still has a negative NPV equal to -$8.71 million. In particular, the NPV for the S2-Solar and S3-Wind improved to reach $37.61 million and $38.73 million, respectively. Conversely, under the most pessimistic estimates, the NPV for the S2-Solar and S3-Wind scenarios deteriorates to approximately -$82.00 million each, while the S5-Wind CCS scenario experiences a significant decline to -$193.64 million. This worsening of NPVs extends the PP to between 12 and 13 years and reduces the ROI to 8–9%. Notably, for the S5-Wind CCS scenario, the NPV projections may shift in response to the European Commission’s 2050 carbon neutrality plan.^52^ The rest of the detailed parameters can be found in Supporting Information Material section 6 in Tables S31–S32.

As a result, the cost of carbon capture could potentially decrease to around 140 €/ton CO_2,_ with future advancements and regulatory changes possibly reducing this cost to between 60 and 140 €/ton CO_2_ (lower than optimiztic scenario). Therefore, with new regulations, this scenario can become economically viable and be used as a competitive advantage.^52^ Therefore, evolving policies and technological advancements could enhance the economic feasibility of this scenario and support its adoption as a viable and competitive solution in the renewable energy sector.

To contextualize our economic assessment, we compared our results with those reported in the literature. One study^20^ highlighted a payback period of 5–6 years with a net present value (NPV) of $32.1 million, while others^27,53^ reported bioethanol selling prices ranging from $0.93 to $2.18 per kg. In our analysis, the NPV for the base case and biomass scenarios (S1-BAU and S4-BAU) ranged from $46 to $54 million, with payback periods of 7–8 years and bioethanol selling prices between $0.80 and $1.41 per kg. These findings align with the methodologies used in the referenced studies, underscoring the economic viability of the proposed biorefinery configurations.

Finally, the breakeven point analysis is conducted to determine the increase in prices of bioproducts across the alternative scenarios needed to achieve parity with the S1-BAU baseline scenario (equal NPV). The results showed that scenario S4-OTP required only an 8.40% increase in product prices to achieve parity with S1-BAU. Conversely, scenarios S2-Solar and S3-Wind, relying solely on renewable energy without carbon capture, faced more significant challenges, requiring 76.95% and 71.98% price increases, respectively.

Lastly, the S5-Wind CCS scenario would require more than doubling the selling price to reach breakeven with the S1-BAU scenario. However, the S5-Wind CCS scenario presents a potential income from selling carbon credits, representing a unique opportunity to mitigate initial costs and achieve economic viability. In recent years, efforts in carbon dioxide removal (CDR) have been increasingly promoted and rewarded through various mechanisms, including direct incentives, tax credits, and the recent EU carbon removal certification framework,^52^ all aimed at incentivizing the sustainable scaling of carbon removal activities. Without selling carbon credits, S5-Wind CCS required a 139.70% price increase to break even. However, the sale of carbon credits at €80 per ton of CO_2_ captured significantly impacts the economic feasibility of the S5-Wind CCS scenario. At this price point, the required increase in selling price for biobased products is reduced to approximately 71.98%. This reduction is notably lower than the price increase needed for the S2-Solar and S3-Wind scenarios. This underscores the potential of carbon credits as a strategic tool to improve the economic feasibility and appeal of CDR projects. By integrating carbon credits, the S5-Wind CCS scenario becomes more economically viable and aligns with broader sustainability goals, offering a compelling investment opportunity. Despite its higher costs, the S5-Wind CCS scenario can leverage these emerging financial mechanisms to generate additional revenue. This includes income from the sale of carbon credits and the production of premium bioproducts, which are valued higher in the market by environmentally conscious consumers. This dual revenue stream not only helps mitigate the additional costs but also enhances the scenario’s overall attractiveness and market competitiveness.

## Environmental Results

### Carbon Footprint Assessment

**Figure 4:**
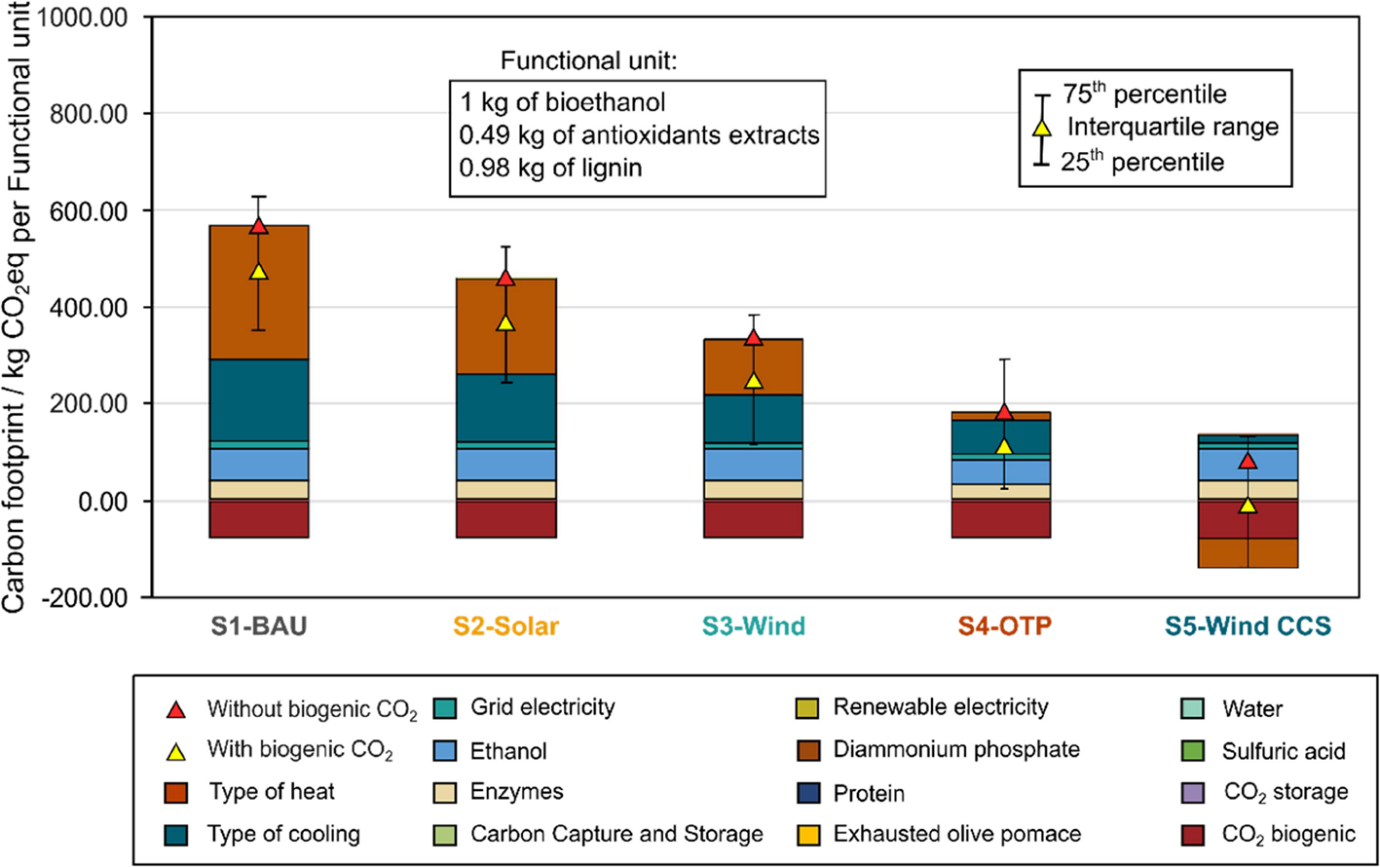
Carbon footprint impact breakdown of the scenarios. Error bars over the deterministic values indicate the variation in carbon footprint considering the uncertainty associated with the inventory data (see details of the sensitivity analysis in the Environmental Assessment section). The yellow triangle represents the results when accounting for the biogenic CO_2_ embodied in the EOP raw material, while the red triangle represents the results without this consideration.

In comparing the carbon footprint of the five scenarios (Figure 4), the analysis revealed that the S1-BAU, the baseline scenario, entails the highest carbon footprint equal to 500.51 kg CO_2_eq/FU cradle-to-gate (Figure 4).

The environmental results are aligned with previous studies showing that the carbon footprint of bioethanol production ranges between 28 to 450 kg CO_2_eq per kg of bioethanol, however, note that the biorefinery also produces the antioxidants extracts and lignin, resulting in at least an increase of around 50% compared to the average range.^54−56^

The scenario S2-Solar, which utilizes solar energy to satisfy partly the electricity and to produce the electrolytic hydrogen for the SNG synthesis, reduces the carbon footprint to 390.36 kg CO_2_eq per FU, primarily due to the renewable nature of the energy source. Scenario S3-Wind further decreased the carbon footprint to 261.59 kg CO_2_eq per FU, benefiting from the high efficiency and low carbon intensity of the wind energy (2.31 × 10^–4^ kg CO_2_eq/kWh). Scenario S4-OTP, which incorporates OTP as a biomass feedstock for electricity and heating, achieved a carbon footprint of 108.32 kg CO_2_eq per FU. Other references also comply with the benefits in this scenario arising from the biogenic nature of the CO_2_ emissions emitted by combusting the OTP.^22,57^ Since the CO_2_ released during combustion is originally captured from the atmosphere during the growth of the olive trees, it is considered carbon neutral, thereby contributing to a lower net carbon footprint. The reduction in the carbon footprint could be even higher if this scenario were coupled with CCS as highlighted by previous works focused on OTP-based biorefineries with CCS.^57^ Finally, the scenario S5-Wind CCS demonstrated the most substantial reduction in carbon footprint, achieving even a negative carbon footprint of −1.05 kg CO_2_eq per FU. This result holds when accounting for the CO_2_ embodied in the bioproducts as well as the CO_2_ geologically sequestered that is captured after the combustion of SNG and from the fermentation (see markers over bars in Figure 4). However, the biogenic CO_2_ released from bioethanol and extractives during their end-use phase contributes to emissions back into the atmosphere. Hence, when considering only the CO_2_ permanently sequestered, the carbon footprint would shift to a positive 78.51 kg CO_2_eq per FU, still representing a substantial reduction compared to the baseline scenario (i.e., 84.31% lower than S1-BAU).

Overall, this scenario effectively sequestered 211.33 kg of CO_2_ per FU, compensating for other emissions associated with the biorefinery operations. Note that this amount of CO_2_ geologically stored includes both the CO_2_ from the fermentation unit (1.98 kg of CO_2_ per FU) as well as the CO_2_ that is captured with DAC to produce SNG is ultimately captured and geologically stored when the latter is combusted to generate the heating requirements (209.35 kg of CO_2_ per FU), ensuring it is kept out of the atmosphere permanently. Overall, the annual permanent CO_2_ removal from the S5-Wind CCS biorefinery is equivalent to 0.41 Mt of CO_2,_ which represents approximately 0.14% of the GHG emitted annually by the Spanish economy.^58^ For the detailed Carbon Footprint results expressed in absolute terms, refer to the Supporting Information Material (section 6 in Tables S33–S38).

The sensitivity analysis demonstrates the high potential variability in carbon footprints (illustrated by the error bars in Figure 4, which depict the uncertainty ranges assessed using Monte Carlo sampling; see Environmental Assessment section for details). For example, scenario S1-BAU shows an uncertainty range for the carbon footprint from 359 to 639 kg CO_2_eq/FU, while scenario S2-Solar ranges from 249 to 535 kg CO_2_eq/FU. Although scenario S5-Wind CCS displays the lowest carbon footprint, the overlap of its uncertainty range (ranging from −142 to 136 CO_2_eq) with those of other scenarios, such as S3-Wind and S4-OTP suggests that the carbon footprints of these scenarios may not differ significantly. Notably, while some scenarios like S3-Wind offer promising reductions in carbon footprint (with a range of 119–389 CO_2_eq), the uncertainties involved must be carefully managed. Implementing such scenarios could yield substantial environmental benefits, but improving data accuracy and reducing uncertainty remains essential for more reliable results.

The breakdown of the carbon footprint in each scenario shown in Figure 4 illustrates the impact of different energy sources and technologies on the overall environmental impact, helping to identify primary drivers and pinpoint areas for further improvements. The main drivers are heating and cooling duties, accounting for around 80–90% of the emissions of the S1-BAU, S2-Solar, and S3-Wind scenarios. In the baseline scenario (S1-BAU), the major contributors are the reliance on nonrenewable energy sources and the emissions associated with fossil natural gas combustion (90%). For the alternative scenarios S2-Solar and S3-Wind, despite the complexity of the SNG production infrastructure involving DAC and electrolytic hydrogen, there is a noticeable reduction in the contribution of the heating and cooling needs compared to the S1-BAU scenarios. In particular, for the S2-Solar, it accounts for 83.41%, while the S3-Wind accounted for 77.12% of the emissions. However, for scenario S4-OTP, the main contributors include the cooling energy needs and bioethanol production (58.26%). Finally, the major drivers of the carbon footprint in the S5-Wind CCS scenario include bioethanol production (64.80 kg CO_2_eq/FU) followed by enzymes (39.63 kg CO_2_eq/FU). Notably, in this scenario, the heating requirements contribute carbon-negative energy intensity (−61.90 kg CO_2_eq/MJ) due to the CO_2_ released from the combustion of SNG (previously captured from the atmosphere with DAC technology) is subsequently geologically stored (with MEA-based CCS technology), offsetting other emissions and contributing negatively to the overall carbon footprint reduction. Hence, 0.41 Mt of CO_2_ would be permanently annually sequestered (211.33 kg per FU) and can be sold as carbon credits.

The S5-Wind-CCS scenario emerges as the most favorable scenario in terms of climate change impacts. The substantial reduction in carbon footprint is attributed to the use of SNG for heating and the capture and storage of CO_2_ emitted during its combustion through the CCS system. This process results in a net negative carbon footprint for the heating requirements, effectively removing more CO_2_ from the atmosphere than is emitted (−2.46 × 10^–2^ kg CO_2_eq/MJ). A detailed breakdown of the carbon footprint drawdown potential is conducted to gain a comprehensive understanding of the carbon footprint for the heating associated with the scenario, considering the production of 1.00 MJ of heating.

**Figure 5:**
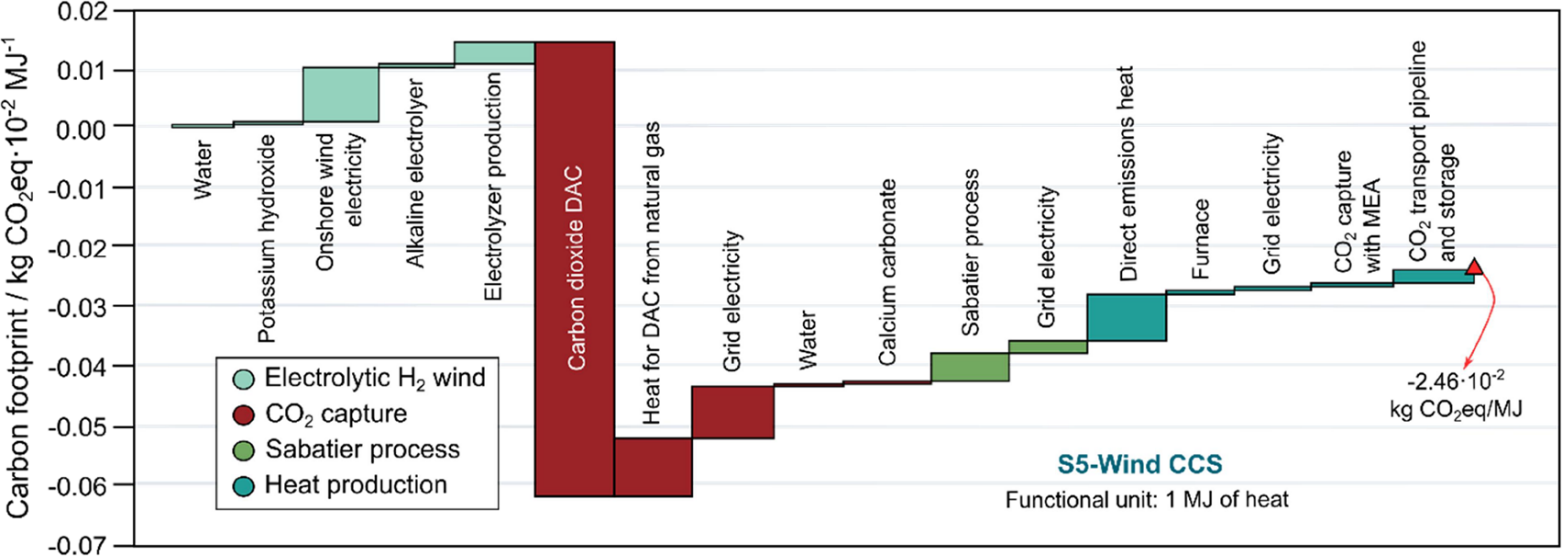
Waterfall diagram illustrating the breakdown of carbon footprint contributors in heating production from SNG, produced from CO_2_ captured and electrolytic H_2_ generated from wind energy, coupled with carbon capture and storage.

The waterfall plot in Figure 5 illustrates the contribution from the raw materials —electrolytic H_2_ and CO_2_ required for the SNG production through the Sabatier process, as well as its use phase and the CCS infrastructure and operation. Note that this scenario assumes that −7.70 × 10^–2^ kg of CO_2_, provided by DAC and modeled as a negative entry of CO_2_ in the biorefinery system, reacts with 9.77 × 10^–3^ kg of H_2_ produced through electrolysis using wind power to produce the SNG. The SNG is then combusted for heating production, and 6.92 × 10^–2^ kg of CO_2_ is captured using the CCS system using MEA at a 90% efficiency, transported via pipeline, and geologically stored. This amount of CO_2_ is physically removed from the atmosphere, compensating for all the life cycle emissions and providing a substantial carbon drawdown of 7.92 × 10^–3^ CO_2_eq per MJ. Further details on the LCA can be found in section 6 of Supporting Information Material Tables S35–S36.

Looking at Figure 5, the production of H_2_ contributes 1.40 × 10^–2^ kg CO_2_eq per MJ, primarily from the substantial amount of wind energy required (9.77 × 10^–3^ kWh per kg of H_2_) and the electrolyzer production, which contributes to 26.35% to the overall footprint of the H_2_ feedstock. This highlights the importance of sourcing low-carbon electricity for the electrolysis process to minimize the carbon footprint associated with H_2_ production.

Regarding the CO_2_ feedstock, it contributes with an overall carbon footprint of −5.80 × 10^–2^ kg CO_2_ eq per kg of CO_2_. This is because the CO2 captured from the atmosphere via DAC is modeled as negative, offsetting the emission associated with the large heating and electricity requirements for the DAC system. By leveraging DAC technology and the CCUS route, the scenario not only mitigates emissions but also actively removes CO_2_ from the atmosphere, offering a dual benefit.

The Sabatier process, used for producing SNG from captured CO_2_ and electrolytic hydrogen, incurs some GHG emissions (+5.08 × 10^–3^ kg CO_2_eq per MJ) due to its energy requirements and other operational factors (Figure 5). Improving the energy efficiency of the Sabatier reaction through advanced catalysts, technological advancements, and optimization of reaction conditions can help reduce the carbon footprint. Moreover, exploring the integration of the Sabatier process with renewable systems to satisfy energy requirements can further lower GHG emissions.

Finally, the combustion of the produced SNG releases 7.92 × 10^–3^ kg CO_2_eq per MJ which corresponds to the uncaptured emissions as the remaining 90% emitted CO_2_ is subsequently captured, transported, and sequestered, which accounts for −6.93 × 10^–2^ kg CO_2_eq per MJ. This step ensures that most of the CO_2_ is permanently removed from the atmosphere, underscoring the effectiveness of CCS in achieving negative emissions.

Despite its environmental benefits, the integration of this CCUS route into the S5-Wind-CCS scenario not only involves addressing the technological and economic challenges associated with H_2_ and CO_2_ production but also requires overcoming logistical barriers. H_2_, being a low-density gas, requires compression or liquefaction for efficient storage and transport, which adds to the overall energy and infrastructure costs.^59^ Similarly, captured CO_2_ must be stored under high pressure or in a supercritical state to facilitate efficient transport, adding another layer of complexity and cost. Although Spain lacks operational CCS facilities, studies highlight promising geological storage potential, including numerous sites in Andalusia (biorefinery location). This analysis assumes a 200 km transport distance and 1000 m storage depth, reflecting typical scenarios for emerging CCS infrastructure.^51^ Investing in technological advancements and strategic planning of robust H_2_ and CO_2_ networks (pipelines or specialized transport vessels) is fundamental to ensure the feasibility of the large-scale implementation of the CCUS route in the long term.

### Occurrence of Burden-Shifting

**Figure 6:**
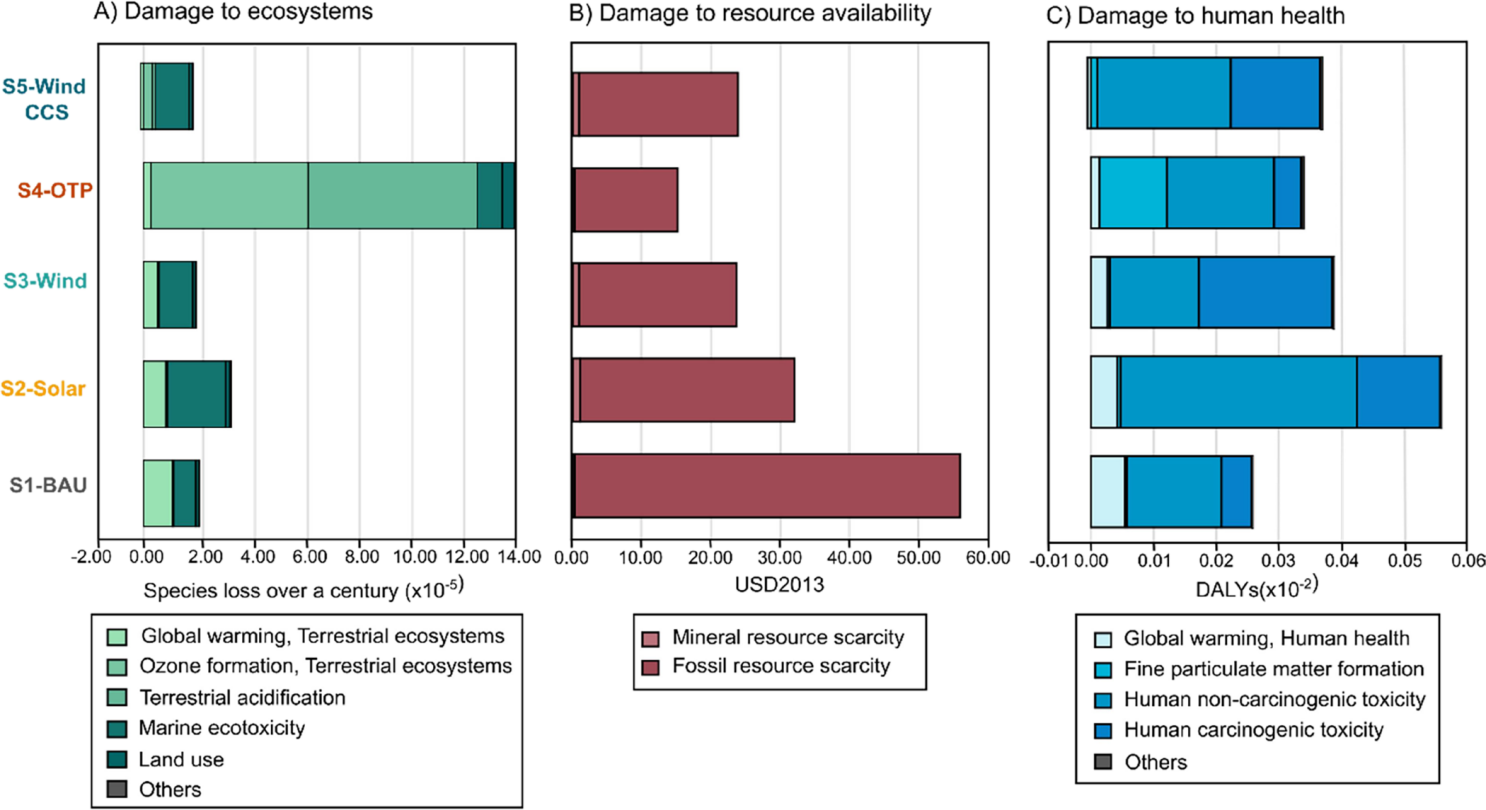
Damage to ecosystems, resource availability, and human health of the five biorefinery scenarios analyzed. (A) Damage to ecosystems expressed in local species loss integrated over time (loss of species over the next 100 years). (B) Damage to resource availability expressed in dollars (USD2013). (C) Damage to human health expressed in disability-adjusted life years (DALYs). Stacked bars present the contributions of individual environmental impact categories to the particular ReCiPe 2016 end point impacts. The category “Other” includes impacts that account for less than 2% of the overall impact.

The analysis then examines the potential occurrence of burden-shifting to identify potential trade-offs of the alternative scenarios. Figure 6 presents a comparative analysis of the five scenarios across the end point categories of the ReCiPe 2016 methodology: damage to ecosystems, damage to resource availability, and damage to human health. Each scenario is represented with stacked bars that depict the contributions of individual environmental impact categories to the overall damage considering the FU.

The damage to ecosystems varies greatly across scenarios. The S4-OTP shows substantially greater impacts (1.39 × 10^–4^ species loss) with high contributions from ozone formation (42%) and terrestrial acidification (45%), which adversely affects biodiversity and habitat integrity. Those impacts are primarily due to the emissions associated with the combustion processes involved in biomass utilization (shown in section 5 from Table S27 from the Supporting Information Material). Specifically, the generation of ozone precursors such as nitrogen oxides (NOx) and volatile organic compounds (VOCs) during the combustion of OTP contributes to elevated levels of ground-level ozone, which is detrimental to both plant and animal life. Furthermore, the release of sulfur dioxide (SO_2_) and NOx leads to acid rain, causing terrestrial acidification that disrupts soil chemistry, damages forests, and negatively impacts aquatic ecosystems by increasing the acidity of water bodies. While the use of OTP leads to damage to ecosystems, it is important to note that these biomass residues would otherwise be underutilized, for example, being burned in the field. This alternative practice not only fails to harness the potential of OTP but also results in emissions of pollutants, contributing to air quality degradation and additional environmental harm. Therefore, the strategic utilization of OTP in controlled combustion processes, coupled with advanced emission control technologies,^22,60,61^ presents an opportunity to mitigate some of these negative environmental side effects while providing a renewable energy source.

Conversely, the BAU scenario and the other alternative scenarios demonstrate much lower ecosystem damage and are of a similar order of magnitude (2.10 × 10^–5^ species loss for S1-BAU, 3.28 × 10^–5^ for S2-Solar, 1.98 × 10^–5^ for S3-Wind and 1.75 × 10^–5^ for S5-Wind CCS), yet the contributors vary greatly across them. For example, in S1-BAU, 51% of the damage is from Global Warming and 40% from Marine ecotoxicity, due to emissions from fossil fuel combustion and the release of pollutants into the marine environment, which result from oil spills, runoff, and other industrial activities associated with the BAU scenario. The marine ecotoxicity impact is more relevant for the S2-Solar, S3-Wind and S5-Wind CCS scenarios, where it represents 66%, 64%, and 73% of the total damage to ecosystems, respectively. This high contribution may be attributed to the life-cycle impacts of these renewable power technologies needed for electrolytic H_2_ production. The installation, decommissioning, and production of solar and wind technologies can contribute to marine ecotoxicity impacts. These effects arise from the extraction and processing of materials, as well as the manufacturing, usage, and disposal of components that release pollutants that may reach marine environments.^62^ The damages to resource availability is a critical metric, reflecting the depletion of both mineral and fossil resources. The S1-BAU shows the highest damage to resource availability (55.75 2013 US$) followed by S2-Solar, S5-Wind CCS, S3-Wind and S4-OTP. In all scenarios, the most significant impact stems from fossil resource scarcity, which underscores the urgent need to reduce fossil fuel dependence throughout the life cycle. In the alternative scenarios, relying on renewables such as solar and wind power, the contribution from mineral resource scarcity is a bit higher (up to 4.50%) due to the intensive mining and processing required for photovoltaic panel production and the wind turbine, including silicon, silver, copper, aluminum and other critical minerals.^62^

Regarding the damage to human health, the S1-BAU shows the lowest damage, equal to 2.56 × 10^–2^ DALYs. Notably, the damage to human health for the S3-Wind, S4-OTP and S5-Wind CCS is 1.51, 1.41, and 1.32 times higher, respectively. This increase is likely due to the logistical complexity associated with SNG production and biomass supply chain, which further complicates the scenarios and adds potential exposure risks. For the S2-Solar, the damage to human health is even more pronounced, doubling compared to S1-BAU. In the S1-BAU scenario, the main contributors are human noncarcinogenic toxicity (59%), global warming, human health (21%) and human carcinogenic toxicity (19%). This indicates that the use of traditional fossil fuels and industrial processes in the BAU scenario primarily affects human health through toxic emissions and greenhouse gases. In contrast, for the alternative scenarios relying on SNG and renewable energy sources such as solar and wind, the contributions are different. Most of the human health impacts in these scenarios come from Human noncarcinogenic toxicity (37%–68%) and Human carcinogenic toxicity (19%–55%), while the impact of Global warming on Human health is negligible, particularly for the S5-Wind CCS. This shift suggests that while renewable technologies reduce GHG, they may still involve harmful pollutants during their production and operational phases, such as the manufacturing stage demand (photovoltaic panels or turbine installation). In the S4-OTP scenario, fine particulate matter formation becomes a primary contributor, accounting for 32% of the overall damage to human health associated with emissions from the biomass combustion processes which can have severe health impacts, including respiratory and cardiovascular diseases. All the detailed results can be found in the Supporting Information Material in section 6 in Table S39.

## Conclusions

This study investigates the technical, economic, and environmental implications of five scenarios of an industrial-scale EOP-based biorefinery to produce bioethanol, antioxidants, and lignin. The analysis showed that alternative scenarios utilizing renewable energy for heat and electricity significantly reduce the carbon footprint compared to the S1-BAU scenario. Notably, the S5-Wind CCS scenario achieves carbon negativity on cradle-to-gate. However, these environmental gains come with increased production costs (between 11%–100%), highlighting the need for technological advancements and scaling-up efforts to enhance the economic viability and competitiveness of biorefineries compared to fossil fuel alternatives. Government and market support are pivotal for the effective implementation of lignocellulosic biorefineries. Enhancing market acceptance of biobased products can be achieved through a comprehensive policy mix, including eco-labeling, taxes on conventional products, and subsidies for sustainable bioproducts.

Implementing premium pricing for these products can accelerate their adoption and market penetration by reflecting their environmental benefits, such as reduced carbon footprint and support for rural economies. Additionally, government financial incentives and CO_2_ removal credits can help offset the higher production costs of bioproducts. Subsidies and funding schemes directed toward research, production, distribution, and consumer purchases can help accelerate innovation in biorefinery processes, improve the efficiency and scalability of production methods, and enhance the distribution networks necessary for the widespread adoption of bioproducts.

On the environmental dimension, the comparison of scenarios underscores the importance of a holistic approach that considers multiple environmental impact categories to avoid burden-shifting. The findings reveal trade-offs between the biorefinery scenarios. While alternative scenarios relying on renewable energy and resources typically demonstrate reduced climate change impacts, they introduce other environmental and health impacts that need careful management. For instance, addressing the logistical challenges and improving biomass and SNG production supply chains can mitigate some associated human health risks. Furthermore, technological advancements, strategic planning, and stringent regulations on pollutant emissions are crucial for minimizing adverse effects and ensuring the sustainable implementation of renewable energy solutions in biorefineries.

In the long term, transitioning away from fossil resources is unavoidable, and biorefineries will play a crucial role in our economy. This work underscores the importance of understanding the role of ETEA in supporting the development of environmentally responsible and economically viable biorefinery systems.

These insights are valuable for policymakers, industry stakeholders, and researchers to make informed decisions, implement effective policies, and drive innovation toward a resilient and sustainable bioeconomy.

## Supporting Information Available

Methodology, process design reactions and simulation, economic and environmental analysis, and results (PDF)
