# Supplementary material for: Uncovering the Techno-Economic and Environmental Implications of a Multiproduct Biorefinery from Exhausted Olive Pomace
Source: ACS Sustain Chem Eng. 2025 Feb 9;13(7):2732–45. doi: 10.1021/acssuschemeng.4c07901 (PMC11866927; doi:10.1021/acssuschemeng.4c07901)
Supplement: Supplementary file 1 — sc4c07901_si_001.pdf [file sc4c07901_si_001.pdf]

Supporting Information

Uncovering the techno-economic and environmental implications of a multiproduct biorefinery from exhausted olive pomace

Deborah Pérez-Almada[1,2], Ángel Galán-Martín[1,2,], María del Mar Contreras[1,2], Juan Miguel Romero-García[1,2], Eulogio Castro[1,2]*

[1]Department of Chemical, Environmental and Materials Engineering, University of Jaén, Campus Las Lagunillas s/n, 23071 Jaén, Spain

[2]Institute of Biorefineries Research (I3B), University of Jaén, Jaén, 23071, Spain

*Corresponding author: galan@ujaen.es

This document includes additional material to the content presented in the main article. Here we report the methodology and design of the simulation, the life cycle inventory and further environmental results and economic analysis.

Number of pages: 61

Number of figures: 6

Number of tables: 39

1. Process design

The component definition of the chemical components used in Aspen Plus for the non-random two-liquid property method was used in the simulations and components in the Aspen simulation to model the liquid and vapor phases according to the following article.[1] Experimental data and results were used as a starting point and the simulation dry matter composition belonged to solid was essentially composed of lignin (38%), xylan (16%), cellulose (16%), ash (11%), and protein (11%).

***Table S1**: Component definition in Aspen Plus*

Component ID	Type	Component name	Alias
WATER	Conventional	WATER	H2O
EXTRACTS	Conventional	DEXTROSE	C6H12O6
GLUCOSE	Conventional	DEXTROSE	C6H12O6
XYLOSE	Conventional	D-XYLOSE	C5H10O5-D2
GALACTOS	Conventional	D-GALACTOSE	C6H12O6-N2
ARABINOS	Conventional	ARABINOSE	C5H10O5-D1
MANNOSE	Conventional	MANNOSE	C6H12O6-N5
MANNITOL	Conventional	D-MANNITOL	C6H14O6-N1
PHENOL	Conventional	PHENOL	C6H6O
ASH	Conventional	SILICON-DIOXIDE	SIO2
PROTEIN	Conventional	LYSINE	C6H14N2O2
GLUCAN	Solid		
XYLAN	Solid		
GALACTAN	Solid		
ARABINAN	Solid		
MANNAN	Solid		
LIGNIN	Solid		
ASHSOLID	Solid	SILICON-DIOXIDE	SIO2
ACETATE	Solid	ACETIC-ACID	C2H4O2-1
PHENOLSO	Solid	PHENOL	C6H6O
PROTESOL	Solid	LYSINE	C6H14N2O2
UNKNOWN	Conventional	DEXTROSE	C6H12O6
5-HMF	Conventional	5-HYDROXYMETHYLFURFURAL	C6H6O3-N5
FURFURAL	Conventional	FURFURAL	C5H4O2
AACETIC	Conventional	ACETIC-ACID	C2H4O2-1
LIGNISOL	Conventional	VANILLIN	C8H8O3-D1
H2SO4	Conventional	SULFURIC-ACID	H2SO4
ETHANOL	Conventional	ETHANOL	C2H6O-2
CO_2	Conventional	CARBON-DIOXIDE	CO_2
DAP	Conventional	DIAMMONIUM-PHOSPHATE	(NH4)2HPO4
BIOMASS	Solid		
LIGNSOL	Conventional	VANILLIN	C8H8O3-D1
ASLIGNIN	Solid	VANILLIN	C8H8O3-D1

AILIGNIN	Solid	VANILLIN	C8H8O3-D1
EXTRATSO	Solid	DEXTROSE	C6H12O6
SUGARS	Conventional	DEXTROSE	C6H12O6
ENZYME	Conventional	LYSINE	C6H14N2O2
AIR	Conventional	AIR	AIR

1.1 Flowsheet process

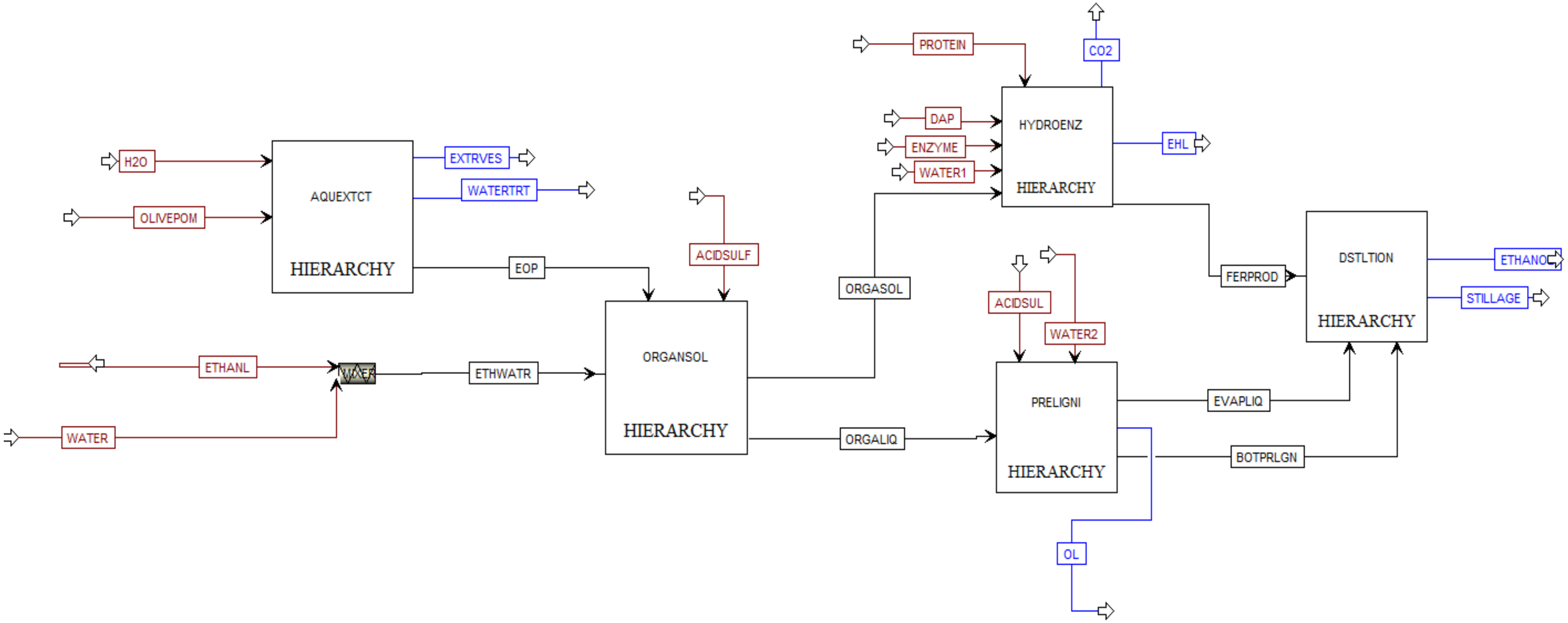

Figure S1: *General flowsheet overview of the multiproduct biorefinery approach.*

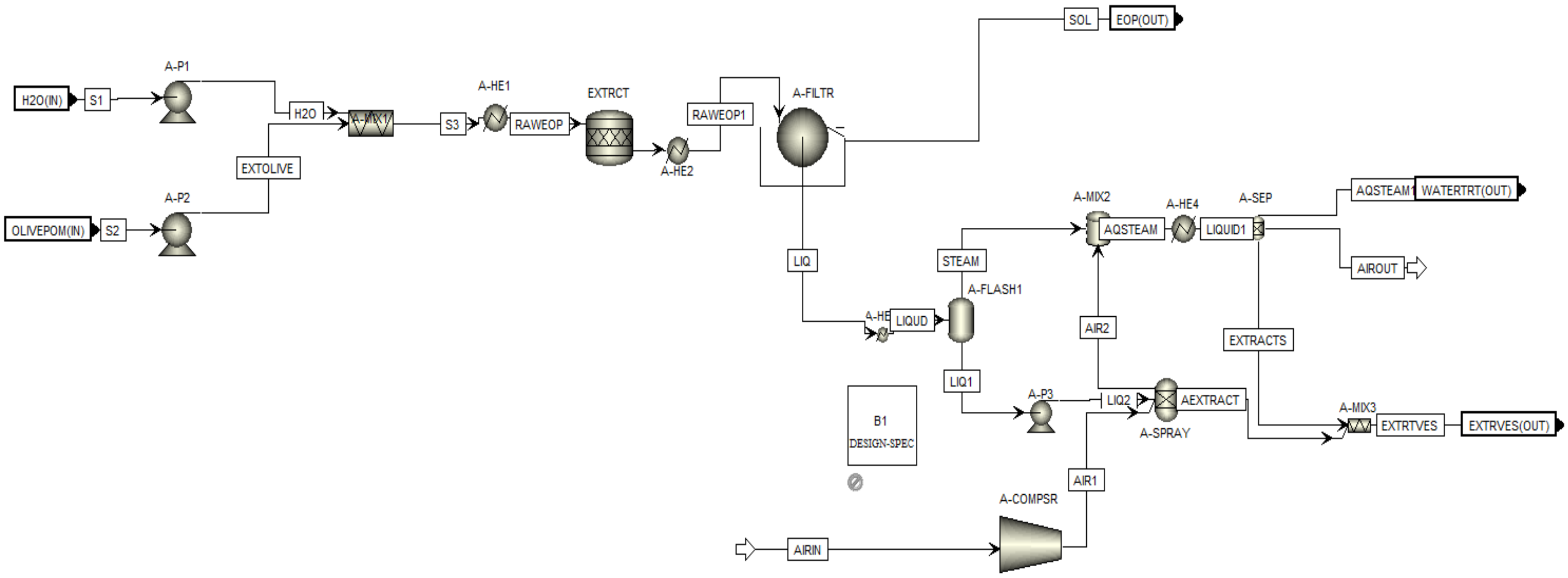

Figure S2: *Flowsheet section of antioxidant extraction and recovery stage shown as AQUEXTCT in Figure S1.*

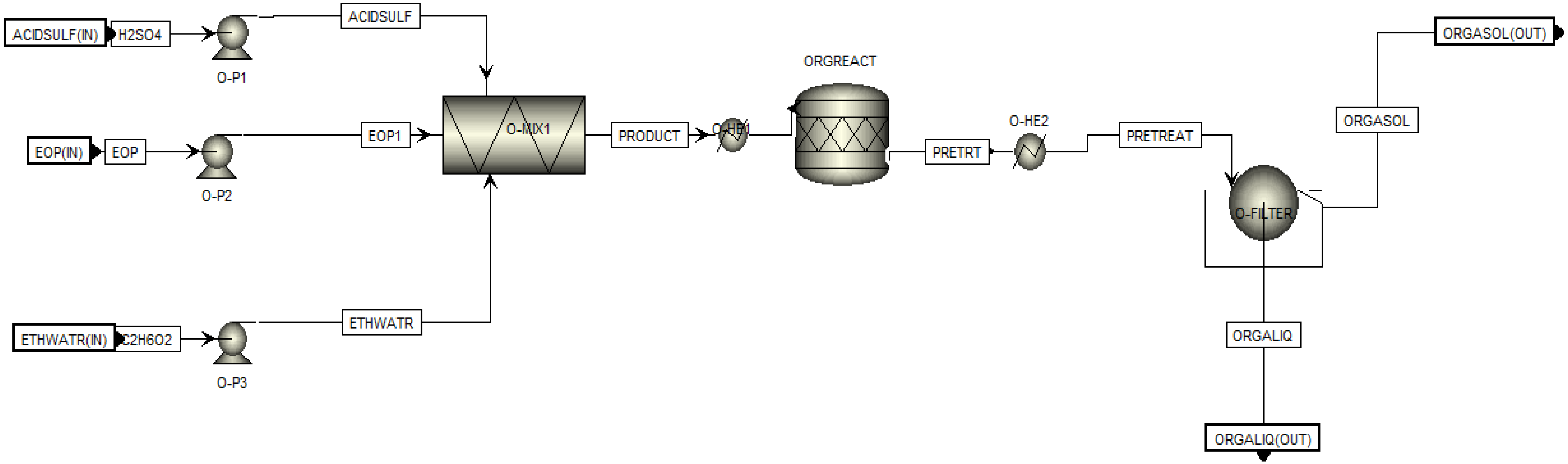

Figure S3*: Flowsheet section of lignocellulosic biomass pretreatment stage shown as ORGANSOL in Figure S1.*

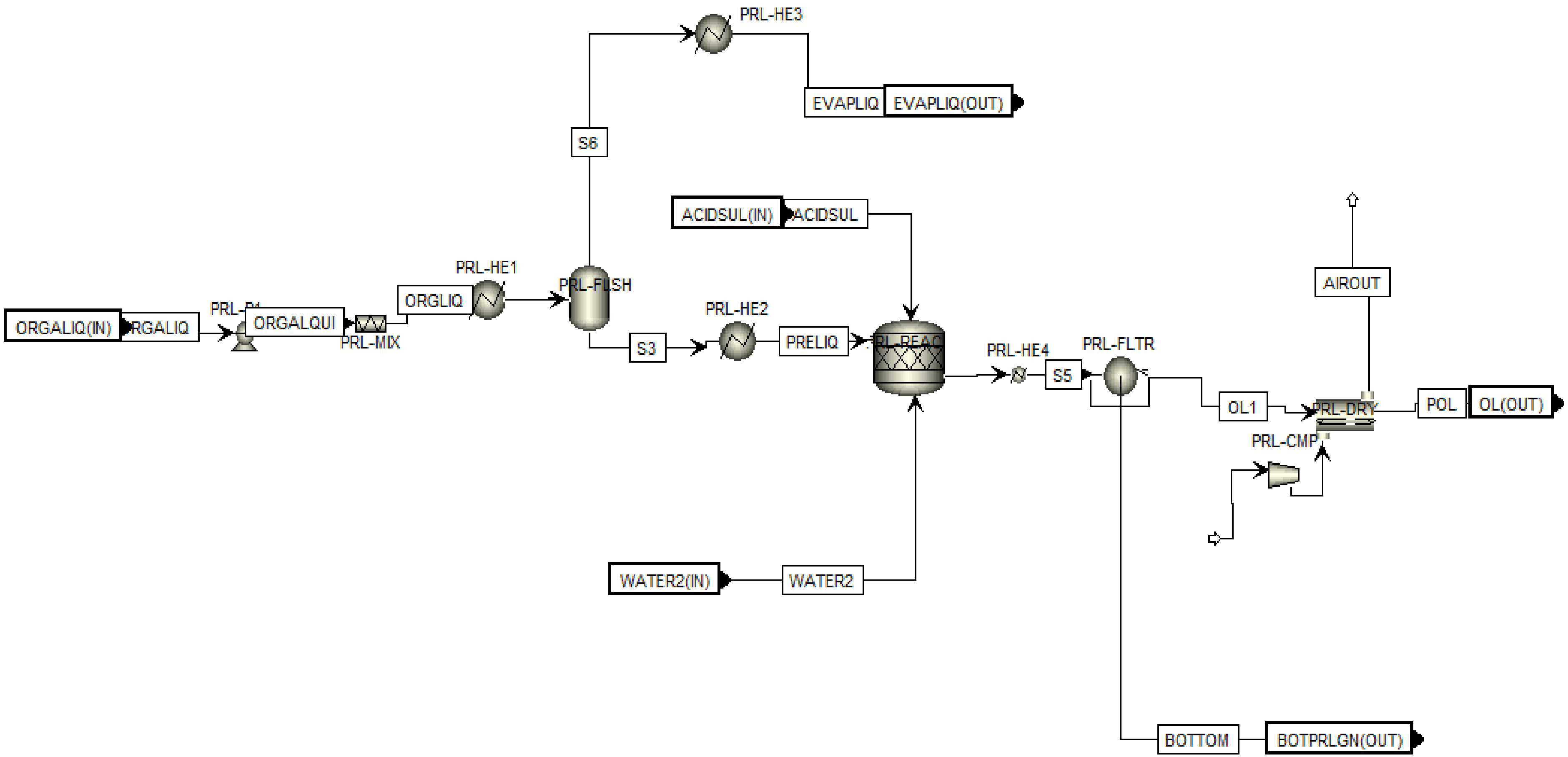

Figure S4: *Flowsheet section of lignin recovery and purification stage shown as PRELIGNI in Figure S1.*

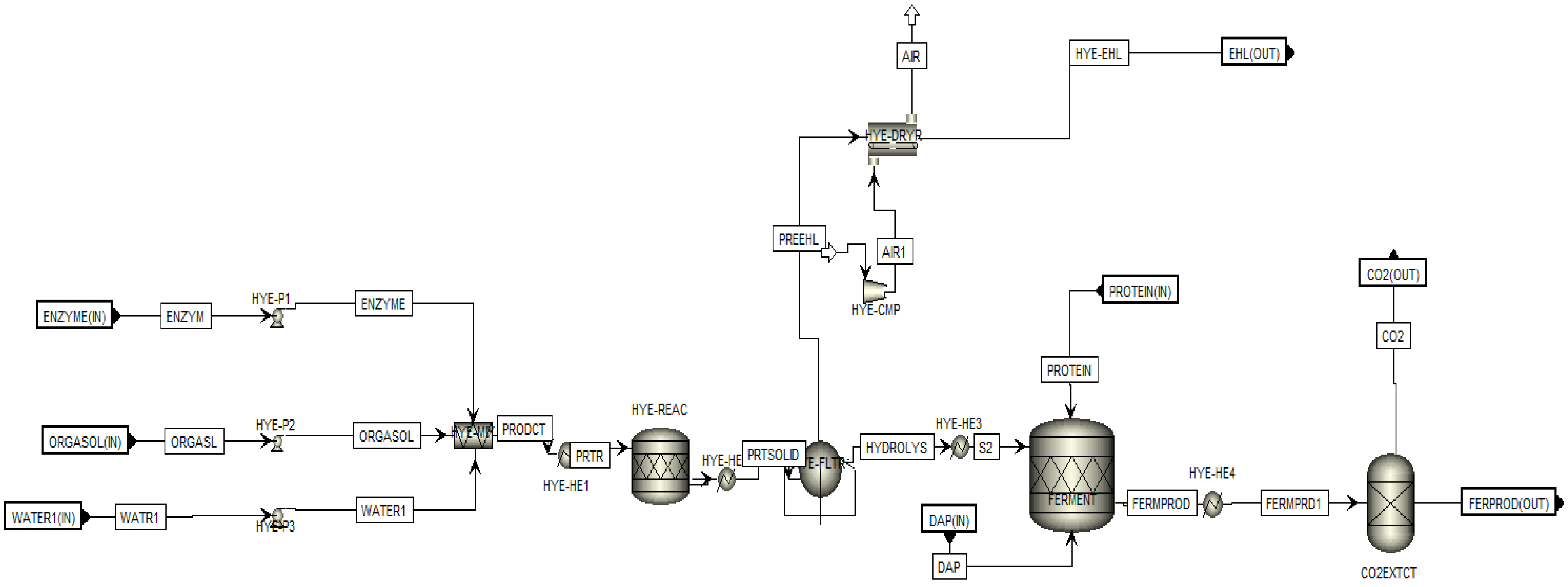

Figure S5: *Flowsheet section of enzymatic hydrolysis and fermentation stage shown as HYDROENZ in Figure S1.*

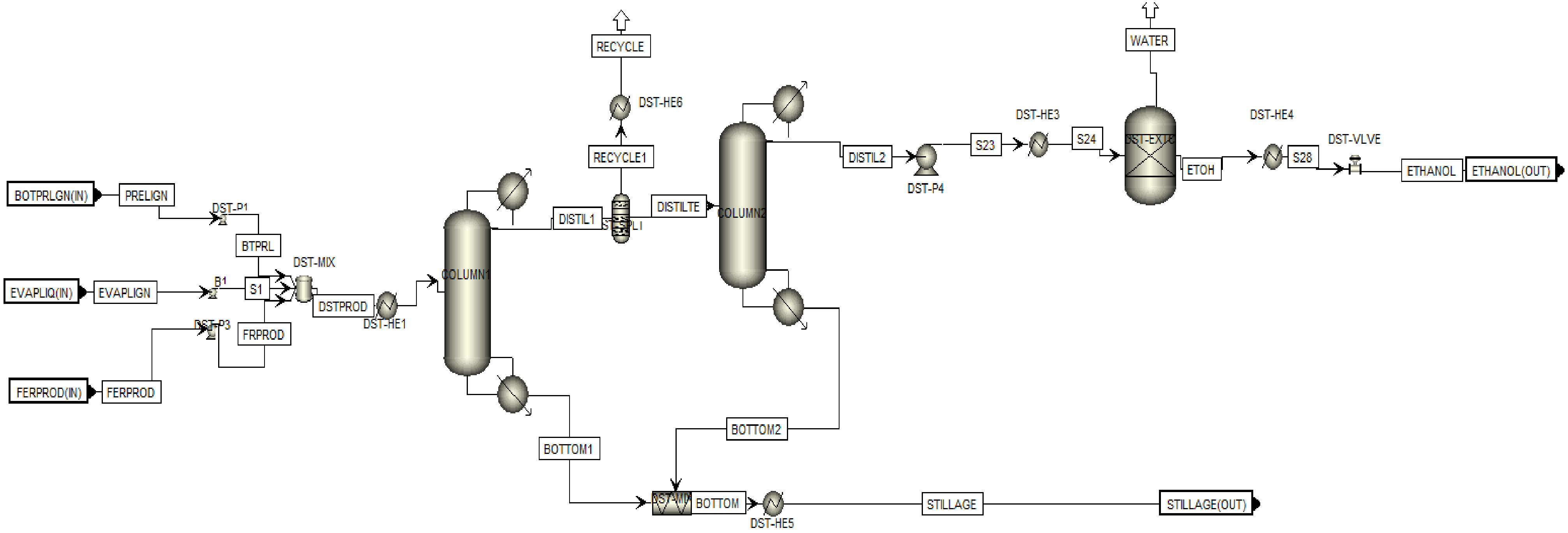

Figure S6: *Flowsheet section of ethanol purification and distillation stage shown as DSTLTION in Figure S1.*

1.2 Reactions considered

The reaction calculations were used following the experimental results from a previous article.[2] A proposed biorefinery approach to obtain antioxidant extracts, lignin and sugars from exhausted olive pomace. Tables S2-S5 include the reactions considered in the antioxidant extraction and recovery, lignocellulosic biomass pretreatment, lignin recovery and purification, enzymatic hydrolysis and fermentation, and ethanol purification and distillation, to give realistic results for a multi-biorefinery scale purpose.

Table S2. *Reactions in reactor: EXTRCT: Antioxidant extraction and recovery stage.*

Rxn No.	Specification type	Units	Fractional conversion	Fractional Conversion of Component	Stoichiometry
1	Frac. conversion	kmol/h	0.21	GLUCAN	WATER + GLUCAN(CIPSD) --> GLUCOSE(MIXED)
2	Frac. conversion	kmol/h	0.21	XYLAN	XYLAN(CIPSD) + WATER --> XYLOSE(MIXED)
3	Frac. conversion	kmol/h	0.33	ARABINAN	WATER + ARABINAN(CIPSD) --> ARABINOS(MIXED)
4	Frac. conversion	kmol/h	$2.80 \cdot 10^{-2}$	LIGNIN	LIGNIN(CIPSD) --> LIGNISOL(MIXED)
5	Frac. conversion	kmol/h	0.29	EXTRACTS	EXTRACTS --> EXTRATSO(CIPSD)
6	Frac. conversion	kmol/h	0.42	PROTESOL	PROTESOL(CIPSD) --> PROTEIN(MIXED)
7	Frac. conversion	kmol/h	0.11	PHENOL	PHENOL --> PHENOLSO(CIPSD)

Table S3. Reactions in the reactor: ORGREACT: Lignocellulosic biomass pretreatment stage.

Rxn No.	Specification type	Units	Fractional conversion	Fractional Conversion of Component	Stoichiometry
1	Frac. conversion	kmol/hr	$1.20\cdot10^{-2}$	GLUCAN	WATER + GLUCAN(CIPSD) --> GLUCOSE(MIXED)
2	Frac. conversion	kmol/hr	0.63	XYLAN	WATER + XYLAN(CIPSD) --> XYLOSE(MIXED)
3	Frac. conversion	kmol/hr	0.50	ARABINAN	ARABINAN(CIPSD) + WATER --> ARABINOS(MIXED)
4	Frac. conversion	kmol/hr	0.26	LIGNIN	LIGNIN(CIPSD) --> LIGNSOL(MIXED)

Table S4. Reactions in reactor: PRL-REACT: Lignin Precipitation and Recovery Stage.

Rxn No.	Specification type	Units	Fractional conversion	Fractional Conversion of Component	Stoichiometry
1	Frac. conversion	kmol/hr	$3.80\cdot10^{-2}$	XYLOSE	XYLOSE --> WATER(MIXED) + XYLAN(CIPSD)
2	Frac. conversion	kmol/hr	0.32	GLUCOSE	GLUCOSE --> WATER(MIXED) + GLUCAN(CIPSD)
3	Frac. conversion	kmol/hr	0.36	ARABINOS	ARABINOS --> WATER(MIXED) + ARABINAN(CIPSD)
4	Frac. conversion	kmol/hr	0.73	LIGNSOL	LIGNSOL --> AILIGNIN(CIPSD)

***Table S4**. Reactions in reactor: HYE-REACT: Enzymatic Hydrolysis and Fermentation stage.*

Rxn No.	Specification type	Units	Fractional conversion	Fractional Conversion of Component	Stoichiometry
1	Frac. conversion	kmol/hr	0.82	GLUCAN	WATER + GLUCAN(CIPSD) --> GLUCOSE(MIXED)
2	Frac. conversion	kmol/hr	0.84	XYLAN	XYLAN(CIPSD) + WATER --> XYLOSE(MIXED)
3	Frac. conversion	kmol/hr	0.72	ARABINAN	ARABINAN(CIPSD) + WATER --> ARABINOS(MIXED)
4	Frac. conversion	kmol/hr	$4.20 \cdot 10^{-3}$	LIGNIN	LIGNIN(CIPSD) --> ASLIGNIN(CIPSD)
5	Frac. conversion	kmol/hr	0.57	LIGNIN	LIGNIN(CIPSD) --> AILIGNIN(CIPSD)
6	Frac. conversion	kmol/hr	0.89	PROTESOL	PROTESOL(CIPSD) --> PROTEIN(MIXED)
7	Frac. conversion	kmol/hr	0.42	LIGNIN	LIGNIN(CIPSD) --> LIGNISOL(MIXED)
8	Frac. conversion	kmol/hr	0.99	ASHSOLID	ASHSOLID(CIPSD) --> ASH(MIXED)
10	Frac. conversion	kmol/hr	1	GALACTAN	WATER + GALACTAN(CIPSD) --> GALACTOS(MIXED)
11	Frac. conversion	kmol/hr	1	MANNAN	WATER + MANNAN(CIPSD) --> MANNOSE(MIXED)
12	Frac. conversion	kmol/hr	1	EXTRATSO	EXTRATSO(CIPSD) --> EXTRACTS(MIXED)
13	Frac. conversion	kmol/hr	1	PHENOLSO	PHENOLSO(CIPSD) --> PHENOL(MIXED)

14	Frac. conversion	kmol/hr	1	ACETATE	ACETATE(CIPSD) --> AACETIC(MIXED)

Table S5. *Reactions in reactor: FERMENTT: Enzymatic Hydrolysis and Fermentation Stage.*

Rxn No.	Specification type	Units	Fractional conversion	Fractional Conversion of Component	Stoichiometry
1	Frac. conversion	kmol/hr	0.96	GLUCAN	GLUCAN(CIPSD) + WATER --> GLUCOSE(MIXED)
2	Frac. conversion	kmol/hr	0.95	GLUCOSE	GLUCOSE --> 2 ETHANOL(MIXED) + 2 CO_2 (MIXED)
3	Frac. conversion	kmol/hr	$2.30 \cdot 10^{-2}$	GLUCOSE	GLUCOSE + 0,018 DAP + 0,3704 PROTEIN --> 2,4 WATER(MIXED) + 6 BIOMASS(CIPSD)
4	Frac. conversion	kmol/hr	0.80	XYLOSE	3 XYLOSE --> 5 ETHANOL(MIXED) + 5 CO_2(MIXED)
5	Frac. conversion	kmol/hr	0.80	ARABINOS	3 ARABINOS --> 5 ETHANOL(MIXED) + 5 CO_2(MIXED)
6	Frac. conversion	kmol/hr	0.90	MANNOSE	MANNOSE --> 2 ETHANOL(MIXED) + 2 CO_2(MIXED)

1.3 Extraction and recovery of the main products: antioxidants extract, ethanol and lignin

The extraction and recovery of the main components were made during all the main stages used in the simulation process. Firstly, the exhausted olive pomace was subjected to the antioxidant extraction and recovery stage. Then, it was vacuum filtered and obtained a solid exhausted olive pomace (EOP) and liquid fraction. The solid fraction was used to continue the simulation process going toward the lignocellulosic biomass pretreatment stage. The liquid fraction was analyzed to determine the content of soluble sugars. The reason behind this was due to its greater biological properties derived from the valuable phenolic compounds.

During the lignocellulosic biomass pretreatment stage, the results from the solid fraction were also filtered to separate the pretreated solid from the pretreated liqueur (aqueous content). Both contents were essential in the recovery of lignin. The pretreated liqueur was filtered and the lignin was recovered by vacuum filtration and called organosolv lignin.

Additionally, the cellulose is hydrolyzed while the pretreated solid is vacuum filtered and the lignin is collected in the Enzymatic hydrolysis and fermentation stage. Lastly, the liquid fraction (enzymatic hydrolysate) from the enzymatic hydrolysis and fermentation stage was fermented and sent to the distillation column (COLUMN1) where the distillate was sent to the last distillation column.

The resulting components from sections 4 and 5 are sent to a distillation column (T-101) where the waxes are obtained as the residue and sent back to the HC reactor. The distillate which contains diesel, kerosene, and gasoline is sent to a second column (T-102). Here, diesel is obtained as the bottom product while the kerosene/gasoline mixture enters the last column (T103), finally splitting into both by-products. The design parameters of the columns can be found in Table S6.

Table S6*. Design parameters of the distillation columns in the separation section in the Ethanol Production Stage*

Columns	Number of stages (feed)	Feed temperature [°C]	Reflux ratio
COLUMN1	5(2)	81.59	2.60
COLUMN2	16(11)	78.23	4.02

2. Simulation results

The essential results from the EOP multi-product biorefinery process design are shown in this section.

Table S7*. Mass flow temperature and pressure from the most essential unit streams of the simulation.*

Unit	Stream	P(bar)	T (ºC)	Mass flow (kg/h)
EXTRCT	RAWEOP	1.01	24.91	129,861.11
EXTRCT	RAWEOP1	1.01	30.00	129,861.11
A-FILTR	RAWEOP1	1.01	30.00	129,861.11
A-FILTR	LIQ	1.01	30.00	117,197.09
A-FILTR	SOL=EOP1	1.01	30.00	12664.02
A-SEP	LIQ	1.01	30.00	339,296.87
A-SEP	WATERTRT	1.01	30.00	112,515.15
A-SEP	EXTRVES	1.01	45.13	4681.94
ORGREACT	SOL=EOP1	1.01	130.00	47855.94
ORGREACT	PRTRT	1.01	82.57	47855.94
O-FILTER	PRTRT	1.01	30.00	47855.94
O-FILTER	ORGALIQ	1.01	30.00	37556.80
PRL-FLSH	ORGALIQ	1.01	85.66	1457.35
PRL-FLSH	EVAPLIQ	1.01	30.00	523.36
PRL-FLSH	PRELIQ	1.01	30.00	933.99

PRL-REAC	PRELIQ	1.01	30.00	21348.67
PRL-REAC	WATER2	1.01	25.00	43848.95
PRL-REAC	ACIDSUL	1.01	30.00	15.88
PRL-REAC	S5	1.01	92.95	65213.50
PRL-FLTR	S5	1.01	30.00	65213.50
PRL-FLTR	BOTPRLGN	1.01	30.00	64311.99
PRL-FLTR	OL1	1.01	30.00	901.52
PRL-DRY	OL1	1.01	30.00	901.52
PRL-DRY	OL	1.01	33.41	495.83
O-FILTER	ORGASOL	1.01	30.00	10299.14
HYE-HE1	ENZYME	1.01	25.00	943.20
HYE-HE1	WATER1	1.01	25.00	16346.05
HYE-HE1	ORGASOL	1.01	30.00	10299.14
HYE-HE1	PRTR	1.01	37.00	27588.38
HYE-REAC	PRTR	1.01	37.00	27588.38
HYE-REAC	PRTSOLID	1.01	93.27	27588.38
HYE-FLTR	PRTSOLID	1.01	30.00	27588.38
HYE-FLTR	PREEHL	1.01	30.00	2425.51
HYE-FLTR	HYDROLYS	1.01	30.00	25162.88
HYE-DRYR	PREEHL	1.01	30.00	2425.51
HYE-DRYR	EHL	1.01	34.96	1334.03
Fermenter	HYDROLYS	1.01	50.00	25162.88
Fermenter	PROTEIN	1.01	25.00	252.53
Fermenter	DAP	1.01	25.00	252.53
Fermenter	FERMPRD1	1.01	41.67	25667.93
CO_2EXTCT	FERMPRD1	1.01	30.00	25667.93
CO_2EXTCT	CO_2	1.01	30.00	541.32
CO_2EXTCT	FERPROD	1.01	30.00	25126.61
COLUMN1	DSTPRD1	1.01	80.00	105,646.72
COLUMN1	DISTIL1	1.01	81.60	35579.59
COLUMN1	BOTTOM1	1.01	100.30	70067.13
COLUMN2	DSTPRD1	1.01	81.60	477.67
COLUMN2	DISTIL2	1.01	78.23	264.46
COLUMN2	BOTTOM2	1.01	99.84	213.22
DST-EXTC	DISTIL1	1.01	150.00	264.46
DST-EXTC	WATER	1.01	150.00	18.08
DST-EXTC	ETHANOL	1.01	150.00	246.38
DST-HE5	BOTTOM	1.01	100.30	70280.35
DST-HE5	STILLAGE	1.01	30.00	70280.35

Table S8*. Mass fraction composition from the most essential unit streams of the simulation.*

Unit	Stream	WATER	EXTRACTS	GLUCOSE	MANNITOL	PHENOL	PROTEIN	LIGNIN	PROTESOL	LIGNSOL	ETHANOL	ASLIGNIN	AILIGNIN
EXTRCT inlet	RAWEOP	0.90	$2.80\cdot10^{-2}$	$2.00\cdot10^{-3}$	$4.00\cdot10^{-3}$	$5.00\cdot10^{-3}$	0	0.02	$6.00\cdot10^{-3}$	0	0	0	0
A-SEP outlet	EXTRVES	0	0.52	$9.90\cdot10^{-2}$	0.11	0.11	$6.70\cdot10^{-2}$	0	$1.70\cdot10^{-2}$	$1.50\cdot10^{-2}$	0	0	0
A-SEP outlet	WATERTRT	1.00	0	0	0	0	0	0	0	0	0	0	0
ORGREACT inlet	ETHWATR	0.48	0	0	0	0	0	0	0	0	0.51	0	0
ORGREACT inlet	ACIDSULF	0	0	0	0	0	0	0	0	0	0	0	0
PRL-REAC inlet	WATER2	1.00	0	0	0	0	0	0	0	0	0	0	0
PRL-REAC inlet	ACIDSUL	0	0	0	0	0	0	0	0	0	0	0	0
PRL-DRY outlet	OL	0	0	0	0	0	0	0	0	0	0	$1.78\cdot10^{-2}$	0.88

HYE-HE1 Inlet	ENZYME	0.80	0	0	0	0	0.20	0	0	0	0	0	0
HYE-HE1 Inlet	WATER 1	1.00	0	0	0	0	0	0	0	0	0	0	0
HYE-DRYR Outlet	EHL	0	0	0	0	0	0	0	$3.55\cdot10^{-2}$	0	0	$5.80\cdot10^{-3}$	0.78
Fermenter Inlet	PROTEIN	0	0	0	0	0	1.00	0	0	0	0	0	0
Fermenter Inlet	DAP	0	0	0	0	0	0	0	0	0	0	0	0
CO_2EXTCT Outlet	CO_2	0	0	0	0	0	0	0	0	0	0	0	0
DST-EXTC Outlet	WATER	1.00	0	0	0	0	0	0	0	0	0	0	0
DST-EXTC Outlet	ETHANOL	$3.80\cdot10^{-3}$	0	0	0	0	0	0	0	0	1.00	0	0
DST-HE5 Outlet	STILLAGE	0.92	$1.61\cdot10^{-2}$	$6.00\cdot10^{-4}$	$3.00\cdot10^{-4}$	$1.10\cdot10^{-3}$	$9.30\cdot10^{-3}$	0	0	$1.06\cdot10^{-2}$	$3.00\cdot10^{-4}$	0	0

3. Heat integration

To optimize and reduce utility energy consumption, the Energy Analyzer tool in Aspen Plus was employed. The results of this analysis are presented in Table S9.

Table S9. *Process utilities demand before and actual heat integration.*

	Before	Actual	Available savings	% of actual
**Total utilities [cal/sec]**	$8.87\cdot10^{10}$	$5.78\cdot10^{10}$	$3.08\cdot10^{10}$	34.79
**Heating utilities [cal/sec]**	$4.47\cdot10^{7}$	$2.93\cdot10^{7}$	$1.54\cdot10^{10}$	34.50
**Cooling utilities [cal/sec]**	$4.40\cdot10^{7}$	$2.85\cdot10^{7}$	$1.54\cdot10^{10}$	35.07

4. Economic analysis

4.1 Capital cost (CAPEX)

The base or purchased cost (Cp^0) data of each piece of equipment is defined by using the equation below:

$$\log(C_P{}^0) = K1 + K2\log(A) + K3[\log(A)]^2 \quad \text{(S1)}$$

The A represents the capacity of the equipment while the equipment type is represented by the constant *K1, K2,* and *K3* along with the maximum and minimum values used in the correlation. For the remaining process equipment, it was developed the pressure factors (Fp) Eq.2 while working under the pressure (P) in a bar. The constants are represented by $C1$, $C2$ and $C3$. The Eq.3 is employed in the case of vessels and distillation columns where the D(m) represents the diameter.

$$\log(Fp) = C1 + C2\log(P) + C3[\log(P)]^2 \quad \text{(S2)}$$

$$Fp_{vessel} = \frac{\frac{P.D}{2[850-0.6(P)]}+0.00315}{0.0063} \quad \text{(S3)}$$

The final module cost (Cbm) considers the material factors (FM) for heat exchangers process vessels and pumps are computed using Eq. 4 The constants of the equation are represented by *B1* and *B2* while the material factor is represented by *Fm*:

$$C_{bm} = Cp^0Fbm = Cp^0(B1 + B2FmFp) \quad \text{(S4)}$$

The parameters from the equations above as well as their respective process units are given in the following table:

Table S10. *CAPEX parameters values (year 2018).*[1]

Equipment	Description	Capacity (units)	*K1*	*K2*	*K3*	*C1*	*C2*	*C3*	*B1*	*B2*	*Fbm*
Compressors	Centrifugal Axial. reciprocating	kW	2.29	1.36	-0.10	0.00	0.00	0.00	-	-	2.80
Dryers	Drum	m^2	4.55	-0.73	0.13				-	-	1.60
Filters	Disc and Drum	m^2	4.81	-0.71	0.04				-	-	1.65
Mixers	Impeller	kW	3.85	-0.30	0.00				-	-	1.38
Heat exchangers	Fixed tube	m^2	4.32	-0.30	0.16	0.04	-0.11	0.08	1.63	1.66	-
Heat exchangers	Kettle reboiler	m^2	4.46	-0.53	0.40	0.00	0.00	0.00	1.63	1.66	-
Heat exchangers	Air cooler	m^2	4.03	0.23	0.05	0.00	0.00	0.00	0.96	1.21	-
Pump	Reciprocating	kW	3.87	0.32	0.12	-0.25	0.26	-0.01	1.89	1.35	-
Reactors	Autoclave	m^3	4.56	-0.70	0.00				-	-	4.00
Reactors	Fermenter	m^3	4.11	-0.47	0.00				-	-	4.00
Towers	Tray and packed	m^3	3.50	0.45	0.11	0.00	0.00	0.00	2.25	1.82	-
Trays	Sieve	m^2	2.99	0.45	0.40	0.00	0.00	0.00	-	-	1.00
Turbines	Axial gas turbines	kW	2.71	1.44	-0.18	0.00	0.00	0.00	-	-	-

[1] *The bare module factor (Fbm) is calculated using Eq.4. The values are directly given in the table in case B1 and B2 are given.*

The cost values were updated to the year 2022 by applying the Chemical Engineering Plant Cost Index (CEPCI) expression in Eq. 5. The parameters used in the equation are shown in Table S11:

***Table S11**: Chemical Engineering Plant Cost Index (CEPCI) used for the simulation.*

**Year**	**CEPCI**
2001	397.00
2005	468.20
2014	576.10
2015	556.80
2016	541.70
2017	567.50
2018	603.10
2019	607.50
2020	596.20
2021	708.00
2022	813.00

$$CAPEX = \frac{CEPCI(new)}{CEPCI(reference)} \sum_{k} Cbm_{k} \quad \forall k \qquad \text{(S5)}$$

For the total Annualization Factor (AF) which is equal to 0.05, the time is represented by 25 years with an interest rate (IR) of 0.05 and is defined by Eq. 6:

$$AF = \frac{IR(IR+1)^{years}}{IR(IR+1)^{years}-1} k \qquad \text{(S6)}$$

The calculations are based on the literature data.[3] Extending the economic analysis, according to the investment parameters demonstrates that the BAU has a positive value of NPV which means the business will be economically appealing with a payback period of approximately 9 years at an internal rate of 5%. Unfortunately, as can be seen from the calculations due to the higher prices S2-Solar, S3-Wind and S5-Wind CCS can only incur losses instead of profit due to the higher prices shown in all scenarios from section 4.2. The rest of the NPV results from the other scenarios are in section 6 in Table S31-S32.

Table S12. *Distribution of capital cost in average scenario (costs are in million $) for 100,000 tonnes per year for the average scenario. The following scheme was made based on the literature data as a guidance*[4–8]

	S1-BAU	S2-Solar	S3-Wind	S4-OTP	S5-Wind CCS
**Total investment**	172.07	192.00	190.72	174.25	208.26
**Contingency**	8.60	9.60	9.54	8.71	10.41
**Fixed capital investment (FCI)**	180.67	201.60	200.25	182.96	218.67
**Total capital investment (TCI)**	363.78	384.71	383.36	366.07	401.78
**NPV values**	54.25	-20.98	-16.12	46.04	-111.64

4.2 Operating Cost (OPEX)

The operating costs are divided into three alternative scenarios which are the average, the best-case, and the worst-case alternative. The central scenario represents our baseline assumptions and average with around 15%-30% lower than the pessimistic scenario and 15%-35% above the optimistic alternative. The operating cost values were used in the table below:

Table S13. *OPEX parameters and costs for bioethanol and antioxidants extracts production of 12.62 ton/h EOP for the central scenario.*

Raw Materials	Mass flow [kg/h]	Average cost [$/kg]
Exhausted olive pomace[9]	12,626.26	$1.50\cdot10^{-2}$
Ethanol[10]	17,902.95	0.50
Sulfuric acid	471.57	0.12
Protein[11]	252.53	$9.50\cdot10^{-2}$
DAP[11]	252.53	0.50
Enzyme[11]	943.20	0.50
Water[11]	77,028.27	$6.70\cdot10^{-3}$
CCS Infrastructure[12,13]	541.32	60.50
DAC[14]	---	238.00 [**$/t$CO_2$**]
H_2 Solar [15–17]	---	6.61
H_2 Wind [15–17]	---	5.57
**Electricity**	**Power [kWh]**	**$/kWh**
Current mix[18]	15,984.58	$9.43\cdot10^{-2}$
Solar PV (20%)[19]	3196.92	$7.50\cdot10^{-2}$
Onshore Wind (25%)[19]	3996.14	$7.75\cdot10^{-2}$
Olive pruning's (100%)[19]	3196.92	0.15
**Utilities**	**Heat flow [kWh]**	**$/kWh**

Heating (Natural gas)[20]	171,440.67	0.12
Cooling (Natural gas)[21,22]	165,862.51	0.13
Heating (Olive pruning's)[19]	171,440.67	0.15
Cooling (Olive pruning's)[19]	165,862.51	0.15
	**Heat flow [MWh]**	**Utility price ($/MWh)**
Heating wind SNG[15–17]	171.44	50.00
Cooling wind SNG [15–17]	165.86	50.00
Heating solar PV+SNG [15–17]	171.44	60.50
Cooling solar PV SNG [15–17]	165.86	60.50

Table S14. *OPEX parameters and costs for bioethanol and antioxidants extract production of 12.62 ton/h EOP for the best-case scenario.*

**Raw Materials**	**Mass flow [kg/h]**	**Average cost [$/kg]**
Exhausted olive pomace	12,626.26	$1.50 \cdot 10^{-2}$
Ethanol	17,902.95	0.47
Sulfuric acid	471.57	$7.00 \cdot 10^{-2}$
Protein	252.53	$9.50 \cdot 10^{-2}$
DAP	252.53	0.37
Enzyme	943.20	0.37
Water	77,028.27	$4.00 \cdot 10^{-5}$
CCS Infrastructure	541.32	35.00
DAC	---	134.00[**$/t$CO_2$**]
H_2 Solar	---	5.67
H_2 Wind	---	4.28
**Electricity**	**Power [kWh]**	**$/kWh**
Current mix	15,984.58	$8.00 \cdot 10^{-2}$
Solar PV (20%)	3196.92	$5.70 \cdot 10^{-2}$
Onshore Wind (25%)	3996.14	$7.20 \cdot 10^{-2}$
Olive pruning's (100%)	3196.92	$9.95 \cdot 10^{-2}$
**Utilities**	**Heat flow [kWh]**	**$/kWh**
Heating (Natural gas)	171,440.67	$9.10 \cdot 10^{-2}$
Cooling (Natural gas)	165,862.51	$9.90 \cdot 10^{-2}$
Heating (Olive pruning's)	171,440.67	$9.95 \cdot 10^{-2}$
Cooling (Olive pruning's)	165,862.51	$9.95 \cdot 10^{-2}$
	**Heat flow [MWh]**	**Utility price ($/MWh)**
Heating wind SNG	171.44	27.00
Cooling wind SNG	165.86	27.00
Heating solar PV+SNG	171.44	29.00
Cooling solar PV SNG	165.86	29.00

Table S15. *OPEX parameters and costs for bioethanol and antioxidants extract production of 12.62 ton/h EOP for the worst-case scenario.*

**Raw Materials**	**Mass flow [kg/h]**	**Average cost [$/kg]**
Exhausted olive pomace	12,626.26	$1.50\cdot10^{-2}$
Ethanol	17,902.95	0.47
Sulfuric acid	471.57	$7.00\cdot10^{-2}$
Protein	252.53	$9.50\cdot10^{-2}$
DAP	252.53	0.37
Enzyme	943.20	0.37
Water	77,028.27	$4.00\cdot10^{-5}$
CCS Infrastructure	541.32	35.00
DAC	---	342[**$/tCO$_2$**]
H_2 Solar	---	7.56
H_2 Wind	---	6.86
**Electricity**	**Power [kWh]**	**$/kWh**
Current mix	15984.58	0.15
Solar PV (20%)	3196.92	0.13
Onshore Wind (25%)	3996.14	$8.30\cdot10^{-2}$
Olive pruning’s (100%)	3196.92	0.21
**Utilities**	**Heat flow [kWh]**	**$/kWh**
Heating (Natural gas)	171,440.67	0.14
Cooling (Natural gas)	165,862.51	0.17
Heating (Olive pruning’s)	171,440.67	0.21
Cooling (Olive pruning’s)	165,862.51	0.21
	**Heat flow [MWh]**	**Utility price ($/MWh)**
Heating wind SNG	171.44	73.00
Cooling wind SNG	165.86	73.00
Heating solar PV+SNG	171.44	92.00
Cooling solar PV+SNG	165.86	92.00

5. LCA Analysis

For the Life Cycle Inventory (LCI) data, the inputs and outputs were organized as presented in the following tables:

Table S16. *Inventory of elementary flows for the production of the biorefinery considering the functional unit of "1 kg de bioethanol + 0.49 kg de antioxidants extracts" together with their respective activity names in Ecoinvent v3.9.1.*

Raw material/ Utilities	Elementary flow	Amount	Activity name
	Outputs/Products:		
**1 kg de bioethanol + 0.49**	Ethanol (kg)	1.00	
	OL (lignin from organosol) (kg)	2.01	
	EHL (lignin from enzymatic hydrolysis)(kg)	5.41	
	Wastewater treatment (m^3)	0.46	
	Antioxidants extracts (kg)[#]	2.05	
	Materials/fuels	**Amount**	**Activity name**
	Exhausted Olive pomace (kg)	51.25	Own activity.
	Water (kg)	788.49	Tap water {RER}| market group for tap water |
	Sulfuric acid(kg)	1.91	Sulfuric acid {RER}| market for sulfuric acid |
	Protein (kg)	1.02	Protein feed. 100% crude {RoW}| ethanol production from whey |
	Diammonium phosphate (kg)	1.02	Diammonium phosphate {RER}| diammonium phosphate production |
	Enzymes (kg)	3.83	Enzymes {RER}| enzymes production |
	Plant	$1.62 \cdot 10^{-4}$	Chemical factory {RER}| chemical factory construction |
	Ethanol (kg)	72.67	Ethanol. without water. in 95% solution state. from fermentation {RoW}| market for ethanol without water in 95% solution state from fermentation |

CO_2 capture and compression plant per kg of ethanol produced (kg)*	1.98	Ref [23]
Carbon dioxide storage from natural gas, post,200 km pipeline, storage 1000 m (kg)*	1.98	Ref [23]
Electricity/heat	**Amount**	**Activity name**
Power (kWh)	64.88	Electricity source varies with the scenario (refer to Table S18)
Heating (GJ)	2.50	Heat source varies with the scenarios (refer to Table S19)
Cooling (GJ)	2.42	Cooling varies with the scenario (refer to Table S20)
Emissions to air		
Carbon dioxide (kg)†	2.20	Carbon dioxide fossil
Carbon dioxide (kg)§	-79.79	Carbon dioxide fossil
Carbon dioxide fossil (kg)*	-1.98	Carbon dioxide fossil
Final waste flows		
Wastewater treatment (m^3)	285.26	Waste unspecified

# For selling purposes we considered a minimum of 10.82% hydroxytyrosol and oleuropein that's why the resulting value.

* These entries were only included for the S5-Wind CCS scenario which includes carbon capture and storage system. It was assumed a 90% capture efficiency from the fermentation unit. The CO_2 from fermentation is almost pure which means the entry will be negative because -2.20x0.90= -1.98 kg.[24]

† This is the CO_2 biogenic emitted during fermentation resulting from the Aspen simulation. Despite being biogenic CO_2, we assumed that this was the negative input of fossil CO_2 in our system. The resulting value is calculated by dividing CO_2 mass flow per the bioethanol (541.32 kg/h / 246.37 kg/h).

§ This is the amount of CO_2 embodied in the EOP raw material. This is calculated considering the carbon content of EOP and the molecular weights of C and CO_2 which is equal to 45.41% CC and moisture content of 6.5% of EOP from a biorefinery approach to obtain antioxidants extract, lignin, and sugars from exhausted olive pomace 51.25 kg EOP x (1 - $6.50 \cdot 10^{-2}$) x 0.45x 44/12 it is is modelled as a negative entry in the system.

To model CO_2 direct air capture (DAC) and the carbon capture storage (CCS) infrastructure the data were taken from literature sources.[23,24] Regarding the CCS infrastructure, we assumed that there would be a capture and storage system that would capture 90% capture efficiency from the fermentation unit. In the LCA, the CO_2 feedstock from DAC was represented as a negative output to represent the removal of ambient CO_2 to meet our plant's input needs. The DAC was used to produce synthetic natural gas by using the Sabatier reaction when reacted with hydrogen derived from different electrical sources (wind and solar). The produced hydrogen derived from photovoltaic solar and onshore wind has been taken from recent literature sources by using a 1 MWe Alkaline electrolyze.[25,26]

The inventory of the EOP was built by modelling the olive oil production from the literature study[27]. Based on the quantity of each product generated, the environmental impact was allocated to all by-products following economic allocation. The economic values for the economic allocation are as follows: virgin olive oil (2.74 $/kg), olive pomace (0.99 $/kg), olive stone (0.07 $/kg) and exhausted olive pomace (0.02 $/kg).[27]

Table S17. *Inventory of elementary flows per unit of raw material and of the olive production process and their respective activity names in Ecoinvent v3.9.1. Data on the olive production was changed in accordance to the Andalusian region olive production which can be found elsewhere.*[27]

Raw material/ Utilities	Elementary flow	Amount	Activity name
**1 kg of olive production**	***Outputs/products:***		
	Exhausted olive pomace (kg)	1887.60	
	Olive stone (kg)	415.50	
	Crude pomace oil (kg)	157.40	
	Virgin olive oil (kg)	1000.00	
	**Material/fuels:**	**Amount**	**Activity name**
	Olives (kg)	4850.87	Olive {ES}| olive production | Cut-off. U
	Current Mix (kWh)	157.25	Electricity. low voltage {ES}| market for electricity. low voltage | Cut-off. U

Cellulose fiber (kg)	1.13	Cellulose fiber {RoW}| market for cellulose fibre | Cut-off. U
Transport. tractor and trailer. agricultural (tkm)	83.55	Transport. tractor and trailer. agricultural {RoW}| market for transport. tractor and trailer. agricultural | Cut-off. U
Petrol. unleaded (kg)	0.02	Petrol. unleaded {Europe without Switzerland}| petrol production. unleaded. petroleum refinery operation | Cut-off. U
Lubricating oil (kg)	0.01	Lubricating oil {RER}| lubricating oil production | Cut-off. U
Cleaning consumables. without water. in 13.6% solution state (kg)	0.30	Cleaning consumables. without water. in 13.6% solution state {GLO}| market for cleaning consumables. without water. in 13.6% solution state | Cut-off. U
Vegetable oil refinery (p)	$5.70 \cdot 10^{-6}$	Vegetable oil refinery {GLO}| market for vegetable oil refinery | Cut-off. U
Tap water (kg)	196.38	Tap water {RER}| market group for tap water | Cut-off. U

Transport. freight. lorry 16-32 metric ton. EURO5 (tkm)	78.55	Transport. freight. lorry 16-32 metric ton. EURO5 {RER}| market for transport. freight. lorry 16-32 metric ton. EURO5 | Cut-off. U
Hexane (kg)	6.13	Hexane {GLO}| market for hexane | Cut-off. U
Vegetable oil refinery (p)	$8.43 \cdot 10^{-7}$	Vegetable oil refinery {GLO}| vegetable oil refinery construction | Cut-off. U

Electricity/heat:	**Amount**	**Activity name**
Current Mix(kWh)	77.04	Electricity. low voltage {ES}| market for electricity. low voltage | Cut-off. U

Table S18. *Power technologies alternatives considered for the multi-biorefinery system in each scenario with each own activity and included inventory.*

Scenario	Electricity alternatives	Activity name
S1-BAU	Current mix	Electricity low voltage {ES}| market for electricity, low voltage |
S2-Solar	Solar photovoltaic	Electricity low voltage {ES}| electricity production, photovoltaic 570kWp open ground installation multi-Si
S3-Wind and S5-Wind CCS	Wind onshore	Electricity high voltage {ES}| electricity production, wind, 1-3MW turbine, onshore |

Scenario	Electricity alternatives	Activity name
S4-OTP	Woodchips	Electricity high voltage {ES}| heat and power co-generation. wood chips 6667 kW state-of-the-art 2014 |

Table S19. *Heating technologies alternatives are considered for the multi-biorefinery system in each scenario with each own activity and included inventory.*

Scenario	Heating alternatives	Activity name
S1-BAU	Heat from natural gas	Heat from steam in chemical industry {RER}| market for heat. from steam in chemical
S2-Solar	Heating from Table S21	Heat, from steam, from Synthetic natural gas from CO_2 from DAC and H_2 solar
S3-Wind	Heating from Table S21	Heat, from steam, from Synthetic natural gas from CO_2 from DAC and H_2 wind
S4-OTP	Heating from Table S26	Heat, from biomass, in chemical industry {RER}| olive pruning production, as energy carrier, RER
S5-Wind CCS	Heating from Table S21	Heat with CCS, Synthetic natural gas from CO_2 DAC and H_2 wind at industrial furnace >100kW |

Table S20. *Cooling technologies alternatives are considered for the multi-biorefinery system in each scenario with each own activity and included inventory.*

Scenarios	Cooling alternatives	Activity name
S1-BAU	Cooling energy	Cooling energy {GLO}| market for cooling energy
S2-Solar	Cooling from Table S29	Cooling energy {ES}| cooling energy, from Synthetic natural gas from CO_2 DAC and H_2 solar PV, at cogen unit with absorption chiller 100kW |
S3-Wind	Cooling from Table S29	Cooling energy {ES}| cooling energy, from Synthetic natural gas from CO_2 DAC and H2 wind, at cogen unit with absorption chiller 100kW | U(MJ)

S4-OTP	Cooling from Table S29	Cooling energy {ES}| cooling energy, from biomass, at cogen unit with absorption chiller 100kW | (MJ),
S5-Wind CCS	Cooling from Table S29	Cooling energy with CCS, cooling energy, from Synthetic natural gas from CO_2 DAC and H2 wind, at cogen unit with absorption chiller 100kW | (MJ),

Table S21*. Inventory of elementary flows for heat from steam with their respective activity names in Ecoinvent v3.9.1.*[25,26]

Raw material/ Utilities	Elementary flow	Amount	Activity name
**1 MJ of heat, from steam, from synthetic natural gas from CO_2 from DAC, and H_2**	***Outputs /products:***		
	Heat, from steam, from synthetic natural gas from CO_2 from DAC and H_2 (MJ)	1.00	
	Materials/fuels:	**Amount**	**Activity name**
	Tap water	$6.18 \cdot 10^{-2}$	Tap water {RER}| market group for tap water |
	Electricity/heat:	**Amount**	**Activity name**
	Grid electricity	$8.08 \cdot 10^{-3}$	Electricity, medium voltage {RER}| market group for electricity, medium voltage |
	Heat, Synthetic natural gas from CO_2 DAC and H_2 at	1.31	See inventory in Table S22

industrial furnace >100kW | (MJ) #		
Emissions to air:	**Amount**	**Activity name**
Water (m^3)	$9.27 \cdot 10^{-6}$	
Emissions to water:	**Amount**	**Activity name**
Water (m^3)	$5.25 \cdot 10^{-5}$	

The heat varies across the scenarios according to the energy source (wind/solar) as shown in Table S19.

Table S22. *Inventory of elementary flows for the heating used in the heating alternatives together with their respective activity names in with each own activity and included inventory.*[25,26] *The H_2 varies according to the electricity source used to produce electrolytic H_2.*

Raw material/ Utilities	Elementary flow	Amount	Activity name
**1 MJ of heat from synthetic natural gas from CO2 from DAC and H_2**	***Outputs /products***		
	Heat from Synthetic natural gas from CO_2 DAC and H_2 at industrial furnace >100kW | (MJ)	1.00	
	Materials/fuels	**Amount**	**Activity name**
	Synthetic natural gas _Sabatier process CO_2 DAC + H_2#	$2.00 \cdot 10^{-2}$	See inventory in Table S23
	Electricity/heat	**Amount**	**Activity name**
	Grid electricity (kWh)	$1.17 \cdot 10^{-3}$	Electricity, low voltage {Europe without Switzerland} | market group for

		electricity, low voltage |
Natural gas production (p)	$2.95 \cdot 10^{-9}$	Industrial furnace, natural gas {RER}| industrial furnace production, natural gas |
Emissions to air	**Amount**	**Activity name**
Acetaldehyde (kg)	$1.05 \cdot 10^{-9}$	
Acetic acid (kg)	$1.58 \cdot 10^{-7}$	
Benzene (kg)	$4.21 \cdot 10^{-7}$	
Benzo(a)pyrene (kg)	$1.05 \cdot 10^{-11}$	
Butane (kg)	$7.37 \cdot 10^{-7}$	
Carbon dioxide, fossil (kg)	$5.89 \cdot 10^{-2}$	
Carbon monoxide, fossil (kg)	$2.21 \cdot 10^{-6}$	
Dinitrogen monoxide(kg)	$1.05 \cdot 10^{-7}$	
Dioxin, 2,3,7,8 Tetrachlorodibenzo -p-(kg)	$3.16 \cdot 10^{-17}$	
Formaldehyde (kg)	$1.05 \cdot 10^{-7}$	
Mercury (II) (kg)	$3.16 \cdot 10^{-11}$	
Methane, fossil (kg)	$2.11 \cdot 10^{-6}$	
Nitrogen oxides (kg)	$1.88 \cdot 10^{-5}$	

PAH, polycyclic aromatic hydrocarbons (kg)	$1.05 \cdot 10^{-8}$
Particulates, < 2.5 um (kg)	$2.11 \cdot 10^{-7}$
Pentane (kg)	$1.26 \cdot 10^{-6}$
Propane (kg)	2.11·10-7
Propionic acid (kg)	$2.11 \cdot 10^{-8}$
Sulfur dioxide (kg)	$5.79 \cdot 10^{-7}$
Toluene (kg)	$2.11 \cdot 10^{-7}$

[#] To calculate the required synthetic natural gas it was necessary to consider that in the main activity of natural gas: $2.72 \cdot 10^{-2}$ m^3 per MJ of heat and it had a natural gas density, high-pressure fossil = 0.73 kg/m^3.Therefore = $2.72 \cdot 10^{-2}$ m^3/MJ x 0.73 kg/m^3 = $2.00 \cdot 10^{-2}$ kg/MJ is going to be the required synthetic natural gas.

Table S23. *Inventory of elementary flows for the production of synthetic natural gas from the Sabatier process for the S2-Solar, S3-Wind, and S-Wind CCS scenarios together with their respective activity names in with each own activity and included inventory.* [25,26]

Raw material/ Utilities	Elementary flow	Amount	Activity name
**1 kg of synthetic natural gas Sabatier process**	***Outputs /products***		
	Synthetic natural gas _Sabatier process CO_2 DAC + H_2 (kg)	1.00	
	Heat waste from Sabatier CO_2 DAC + H_2 (kWh)	3.01	
	Materials/fuels	**Amount**	**Activity name**

Carbon dioxide DAC[#]	2.94 x 50.1	See inventory in Table S24
H_2 electrolytic[*]	0.51 x 50.1	See inventory in Table S25
Electricity/heat	**Amount**	**Activity name**
Grid electricity (kWh)	0.33	Electricity, low voltage {ES}| market for electricity, low voltage |
Emissions to air	**Amount**	**Activity name**
Hydrogen (kg)	0.01	
Carbon Dioxide (kg)	0.19	

[#]For $1.00 \cdot 10^{-3}$ GJ of Heat from synthetic natural gas from CO_2 from DAC and H_2 solar PV at industrial furnace we have $2.00 \cdot 10^{-2}$ of Synthetic natural gas _Sabatier process CO_2 DAC + H_2 Solar PV open-ground (kg) and the 2.50 GJ of Heat from the simulation. As a result, ($2.00 \cdot 10^{-2}$ kg/GJ x 2.50 GJ)/ $1.00 \cdot 10^{-3}$GJ=50.1 kg. This means for 1MJ the Synthetic natural gas Sabatier photovoltaic process will have 50.1kg. Therefore, for Carbon dioxide, DAC would have to be multiplied by 2.94 and the final result would be 147.24kg.
[*]For hydrogen electrolytic, the 50.1 kg would have been multiplied by 0.51 and the final result would be 23.35kg. The hydrogen can be electrolytic wind or solar depending on the scenario.

Table S24. *Inventory of elementary flows for the CO_2 for direct air capture (DAC) used in the heating and cooling alternatives together with their respective activity names in Ecoinvent v3.9.1.* [25,26]

Raw material/ Utilities	Elementary flow	Amount	Activity name
**1 kg of Carbon dioxide**	***Outputs/products***		
	Carbon dioxide DAC (kg)	1.00	
	Electricity/heat	**Amount**	**Activity name**

	Heat, district or industrial, natural gas (MJ)	5.25	Heat, district or industrial, natural gas {ES}| heat and power co-generation, natural gas, combined cycle power plant, 400MW electrical |
	Grid electricity (kWh)	0.37	Electricity, low voltage {ES}| market for electricity, low voltage |
	Water (kg)	3.11	Tap water {RER}| market group for tap water |
	Calcium carbonate (kg)	0.02	Calcium carbonate, precipitated {RER}| calcium carbonate production, precipitated |
	Emissions to air	**Amount**	**Activity name**
	Carbon dioxide (kg)	-1.00	

Table S25. *Inventory of elementary flows for the electrolytic hydrogen for S2-Solar, S3-Wind and S5-Wind CCS scenario used in the heating and cooling alternatives together with their respective activity names in Ecoinvent v3.9.1.*[25,26,28]

Raw material/ Utilities	Elementary flow	Amount	Activity name
**1 kg hydrogen electrolytic from different sources**	***Outputs /products***		
	Hydrogen electrolytic (kg)	1.00	

Materials/fuels	**Amount**	**Activity name**
Occupation (m^{2a})	$7.75 \cdot 10^{-4}$	Occupation, industrial area
Transformation (m$^{2)}$	$2.82 \cdot 10^{-5}$	Transformation, from industrial area
Transformation (m^{2})	$2.82 \cdot 10^{-5}$	Transformation, to industrial area
Resources	**Amount**	**Activity name**
Water deionised (kg)	14.00	Water, deionised {Europe without Switzerland}| market for water, deionised |
Potassium hydroxide (kg)	$3.70 \cdot 10^{-3}$	Potassium hydroxide {RER}| potassium hydroxide production |
Electricity/heat	**Amount**	**Activity name**
Electricity source varies across scenarios (kWh)	51.82	See inventory in Table S18
Electrolyzer production, 1MWe, AEC, Stack (p)	$9.39 \cdot 10^{-7}$	Ref [25,26,2827,30]
Alkaline electrolyzer (p)	$2.35 \cdot 10^{-7}$	Ref [25,26,2827,30]
Emissions to air	**Amount**	**Activity name**
Oxygen (kg)	8.02	

Table S26. *Inventory of elementary flows for the heating from the S4-OTP scenario together with their respective activity names in with each own activity and included inventory.*[29]

Raw material/ Utilities	Elementary flow	Amount	Activity name
	Outputs /products:		
**1 MJ of heat from olive tree pruning**	Heat, from olive tree pruning, in chemical industry {RER}| olive pruning production, as energy carrier, ES test (MJ)	1.00	
	Materials/fuels:	**Amount**	**Activity name**
	Tap water	$6.18 \cdot 10^{-2}$	Tap water {RER}| market group for tap water |
	Electricity/heat:	**Amount**	**Activity name**
	Grid electricity	$8.08 \cdot 10^{-3}$	Electricity, medium voltage {RER}| market group for electricity, medium voltage |
	Heat, district or industrial, other than natural gas {ES}| heat and power co-generation, olive prunnings, 6667 kW, state-of-the-art 2014 | (MJ)	1.31	See inventory in Table S27
	Emissions to air:	**Amount**	**Activity name**
	Water (m^3)	$9.27 \cdot 10^{-6}$	
	Emissions to water:	**Amount**	**Activity name**
	Water, (m^3)	$5.25 \cdot 10^{-5}$	

The inventory "Heat and power co-generation, wood chips, 6667 kW, state-of-the-art" was taken from Ecoinvent v3.9.1 which represents the heating of a biomass plant of woodchips and the raw material was changed to olive three pruning's (OTP). The OTP has changed accordingly with its energy content and direct emissions according to the literature reference[29].

Table S27*. Inventory of elementary flows for the heating from the S4-OTP scenario, together with their respective activity names in with each own activity and included inventory.*

Raw material/ Utilities	Elementary flow	Amount	Activity name
	Outputs /products:		
**1 MJ of heat, from olive tree pruning**	Heat, district or industrial, other than natural gas {ES}| heat and power co-generation, olive pruning's, 6667 kW, state-of-the-art 2014 |,(MJ)	1.00	
	Materials/fuels:	**Amount**	**Activity name**
	Ammonia, anhydrous (kg)	$6.15 \cdot 10^{-9}$	Ammonia, anhydrous, liquid {RER}| market for ammonia, anhydrous, liquid |
	Chemical, organic (kg)	$4.39 \cdot 10^{-6}$	Chemical, organic {GLO}| market for chemical, organic |
	Chlorine, liquid (kWh)	$2.46 \cdot 10^{-7}$	Chlorine, liquid {RER}| market for chlorine, liquid |
	Dust collector, electrostatic precipitator, for industrial use (p)	$3.90 \cdot 10^{-10}$	Dust collector, electrostatic precipitator, for industrial use {GLO}| market for dust collector, electrostatic precipitator, for industrial use |

Lubricating oil (kg)	$2.46 \cdot 10^{-6}$	Lubricating oil {RER}| market for lubricating oil |
NOx retained (kg)	$6.00 \cdot 10^{-5}$	NOx retained, by selective catalytic reduction {GLO}| market for NOx retained, by selective catalytic reduction |
Sodium chloride, powder (kg)	$3.07 \cdot 10^{-6}$	Sodium chloride, powder {GLO}| market for sodium chloride, powder |
Water (kg)	$5.90 \cdot 10^{-4}$	Water, decarbonized {ES}| market for water, decarbonized |
Olives (kg)[#]	$3.48 \cdot 10^{-2}$	Olive {ES}| olive production |, measured as dry mass |
Electricity/heat:	**Amount**	**Activity name**
Furnace, wood chips, with silo (p)	$3.90 \cdot 10^{-2}$	Furnace, wood chips, with silo, 5000kW {GLO}| market for furnace, wood chips, with silo, 5000kW |
Heat and power co-generation unit, organic Rankine cycle, (p)	$3.90 \cdot 10^{-2}$	Heat and power co-generation unit, organic Rankine cycle, 1000kW electrical {GLO}| market for heat and power co-generation unit, organic Rankine cycle, 1000kW electrical |
Emissions to air:	**Amount**	**Activity name**
Carbon monoxide (kg)*	134.40	
Sulfur dioxide (kg)*	0.15	
Total Organic Carbon (kg)*	0.36	
Nitrogen oxides (kg)*	44.15	

Sodium (kg)*	$6.68\cdot10^{-2}$	
Magnesium (kg)*	$2.41\cdot10^{-2}$	
Aluminum (kg)*	$8.36\cdot10^{-2}$	
Potassium (kg)*	$9.76\cdot10^{-2}$	
Calcium (kg)*	$5.55\cdot10^{-2}$	
Chromium compounds (kg)*	$1.35\cdot10^{-2}$	
Manganese compounds (kg)*	$5.61\cdot10^{-4}$	
Iron compounds (kg)*	$1.91\cdot10^{-2}$	
Nickel compounds (kg)*	$1.80\cdot10^{-2}$	
Copper compounds (kg)	$3.37\cdot10^{-3}$	
Zinc compounds (kg)*	$7.85\cdot10^{-3}$	
Strontium (kg)*	134.40	
Waste to Treatment:	**Amount**	**Activity name**
Municipal solid waste (kg)	$2.46\cdot10^{-6}$	Municipal solid waste {ES}| market for municipal solid waste |
Waste mineral oil (kg)	$2.46\cdot10^{-6}$	Waste mineral oil {Europe without Switzerland} | market for waste mineral oil |
Wastewater, average (kg)	$5.90\cdot10^{-7}$	Wastewater, average {Europe without Switzerland} | market for wastewater, average |

	Wood ash mixture (kg)	$3.97\cdot10^{-4}$	Wood ash mixture, pure {Europe without Switzerland} | market for wood ash mixture, pure |

[#] We considered the LHV of olive pruning:15.8 MJ/kg (dry mass) to calculate the olive pruning and a burning efficiency of 15%. Therefore, to calculate the required mass for the olive pruning it was necessary to use the following equation: Olive pruning mass (1-0.15)/15.8 MJ/kg which is equal to $3.48\cdot10^{-2}$ MJ/kg.
[*] Based on the literature reference we take the direct combustion emissions into account.[30]

Table S28. *Inventory of elementary flows for the heating from the S5-Wind CCS scenario with their respective activity names in with each own activity and included inventory.*[25,26]

Raw material/ Utilities	Elementary flow	Amount	Activity name
	Outputs /products:		
**1 MJ of heat with CCS from synthetic natural gas from CO_2 from DAC and H_2 wind**	Heat with CCS, Synthetic natural gas from CO_2 DAC and H_2 wind at industrial furnace >100kW | (MJ)	1.00	
	Materials/fuels:	**Amount**	**Activity name**
	Synthetic natural gas _Sabatier process CO_2 DAC + H_2	$2.00\cdot10^{-2}$	See inventory in Table S23
	Carbon dioxide, captured from natural gas, post, 200km pipeline, storage 1000m [#]	$5.30\cdot10^{-2}$	Ref [23]
	Electricity/heat:	**Amount**	**Activity name**
	Grid electricity (kWh)	$1.17\cdot10^{-3}$	Electricity, low voltage {Europe without Switzerland} | market group for

		electricity, low voltage |
Natural gas production (p)	$2.95\cdot10^{-9}$	Industrial furnace, natural gas {RER}| industrial furnace production, natural gas |
Emissions to air:	**Amount**	**Activity name**
Acetaldehyde (kg)	$1.05\cdot10^{-9}$	
Acetic acid (kg)	$1.58\cdot10^{-7}$	
Benzene (kg)	$4.21\cdot10^{-7}$	
Benzo(a)pyrene (kg)	$1.05\cdot10^{-11}$	
Butane (kg)	$7.37\cdot10^{-7}$	
Carbon dioxide, fossil (kg)	$5.89\cdot10^{-2}$	
Carbon monoxide, fossil (kg)	$2.21\cdot10^{-6}$	
Dinitrogen monoxide (kg)	$1.05\cdot10^{-7}$	
Dioxin, 2,3,7,8 Tetrachlorodibenzo-p-(kg)	$3.16\cdot10^{-17}$	
Formaldehyde (kg)	$1.05\cdot10^{-7}$	
Mercury (II) (kg)	$3.16\cdot10^{-11}$	
Methane, fossil (kg)	$2.11\cdot10^{-6}$	
Nitrogen oxides (kg)	$1.88\cdot10^{-5}$	
PAH, polycyclic aromatic hydrocarbons (kg)	$1.05\cdot10^{-8}$	

	Particulates, < 2.5 um (kg)	$2.11 \cdot 10^{-7}$	
	Pentane (kg)	$1.26 \cdot 10^{-6}$	
	Propane (kg)	$2.11 \cdot 10^{-7}$	
	Propionic acid (kg)	$2.11 \cdot 10^{-8}$	
	Sulfur dioxide (kg)	$5.79 \cdot 10^{-7}$	
	Toluene (kg)	$2.11 \cdot 10^{-7}$	
	Carbon dioxide, fossil (kg)	$-5.30 \cdot 10^{-2}$	

[#]We are assuming that there will be a capture and storage system that will capture 90% and as a result, we will have $-0.90 \times 5.89 \cdot 10^{-2}$ kg = $-5.30 \cdot 10^{-2}$ kg.

Table S29. *Inventory of elementary flows for the cooling alternative from scenarios and their respective activity names in each activity and included inventory.*[25,26]

Raw material/ Utilities	Elementary flow	Amount	Activity name
	Outputs products:		
**1MJ of cooling**	Cooling source varies (MJ)[#]	1.00	
	Materials/fuels:	**Amount**	**Activity name**
	Water, decarbonized (kg)	$2.51 \cdot 10^{-2}$	Water, decarbonised {BR}| market for water, decarbonised |
	Water, decarbonized (kg)	$6.54 \cdot 10^{-2}$	Water, decarbonised {CA}| market

		for water, decarbonised |
Water, decarbonized (kg)	$2.64 \cdot 10^{-1}$	Water, decarbonised {CN}| market for water, decarbonised |
Water, decarbonized (kg)	$6.44 \cdot 10^{-2}$	Water, decarbonised {DE}| market for water, decarbonised |
Water, decarbonized (kg)	$1.30 \cdot 10^{-2}$	Water, decarbonised {ES}| market for water, decarbonised |
Water, decarbonized (kg)	$4.27 \cdot 10^{-2}$	Water, decarbonised {FR}| market for water, decarbonised |
Water, decarbonized (kg)	$2.36 \cdot 10^{-3}$	Water, decarbonised {GB}| market for water, decarbonised |
Water, decarbonized (kg)	$3.36 \cdot 10^{-2}$	Water, decarbonised {IN}| market for water, decarbonised |
Water, decarbonized (kg)	$4.17 \cdot 10^{-1}$	Water, decarbonised {RoW}| market for water, decarbonised |

Water, decarbonized (kg)	$7.83 \cdot 10^{-2}$	Water, decarbonised {RU}| market for water, decarbonised |
Water, decarbonized (kg)	$4.91 \cdot 10^{-1}$	Water, decarbonised {US}| market for water, decarbonised |
Water, decarbonized (kg)	$3.21 \cdot 10^{-3}$	Water, decarbonised {ZA}| market for water,
Electricity/heat:	**Amount**	**Activity name**
Absorption chiller (p)	$1.39 \cdot 10^{-7}$	Absorption chiller, 100kW {GLO}| market for absorption chiller, 100kW |
Grid electricity (kWh)	$1.92 \cdot 10^{-4}$	Electricity, low voltage {ES}| market for electricity, low voltage |
Grid electricity (kWh)	$2.67 \cdot 10^{-3}$	Electricity, low voltage {Europe without Switzerland}| market group for electricity, low voltage |
Grid electricity (kWh)	$3.50 \cdot 10^{-5}$	Electricity, low voltage {NZ}| market for electricity, low voltage |
Grid electricity (kWh)	$5.66 \cdot 10^{-4}$	Electricity, low voltage {RAF}| market group for

		electricity, low voltage |
Grid electricity (kWh)	$1.08 \cdot 10^{-2}$	Electricity, low voltage {RAS}| market group for electricity, low voltage |
Grid electricity (kWh)	$1.12 \cdot 10^{-3}$	Electricity, low voltage {RLA}| market group for electricity, low voltage |
Grid electricity (kWh)	$3.85 \cdot 10^{-3}$	Electricity, low voltage {RNA}| market group for electricity, low voltage |
Grid electricity (kWh)	$7.86 \cdot 10^{-4}$	Electricity, low voltage {RU}| market for electricity, low voltage |
Heating source varies (MJ)[#]	1.67	See inventory in Table S18
Emissions to air:	**Amount**	**Activity name**
Heat waste (MJ)	$7.20 \cdot 10^{-2}$	
Water (m^3)	$5.81 \cdot 10^{-4}$	
Emissions to water:	**Amount**	**Activity name**
Water, (m^3)	$9.19 \cdot 10^{-4}$	

[#] The cooling and heating source will vary according to the type of scenario (solar/wind). For scenarios S2-Solar, S3-Wind, and S5-Wind CCS will be "Heat, central or small scale with from synthetic natural gas from CO_2 from DAC and H_2" as shown in table S32. However, for S4-OTP the heating activity was taken from Ecoinvent v3.9. and is called "Heat, district or industrial, other than natural gas {ES}| heat and power co-generation, olive pruning's, 6667 kW, state-of-the-art 2014 | (MJ)".

Table S30. *Inventory of elementary flows for the heating source used for cooling from the S2- Solar, S3-Wind, and S5-Wind CCS scenarios together with their respective activity names in with each own activity and included inventory.*[25,26]

Raw material/ Utilities	Elementary flow	Amount	Activity name
	Outputs /products:		
**1MJ of Heat, central or small scale with synthetic natural gas from CO_2 from DAC and H_2**	Heat, central or small-scale, Synthetic natural gas CO_2 DAC and H_2 (MJ)	1.00	
	Materials/fuels:	**Amount**	**Activity name**
	Lubricating oil (kg)	$1.23 \cdot 10^{-5}$	Lubricating oil {RoW}| market for lubricating oil |
	Synthetic natural gas _Sabatier process CO_2 DAC + H_2 (kg)	$8 \cdot 10^{-3}$	See inventory in Table S23
	Pipeline, natural gas, low pressure distribution network (km) #	$1.51 \cdot 10^{-9}$	Pipeline, natural gas, low pressure distribution network {GLO}| market for pipeline, natural gas, low pressure distribution network |
	Natural gas, burned in gas turbine (MJ)*	$4.64 \cdot 10^{-4}$	Natural gas, burned in gas turbine {US}| natural gas, burned in gas turbine |

Electricity/heat:	**Amount**	**Activity name**
Heat and power co-generation unit (p)	$2.05\cdot10^{-9}$	Heat and power co-generation unit, 160kW electrical, common components for heat + electricity {GLO}| market for heat and power co-generation unit, 160kW electrical, common components for heat + electricity |
Heat and power co-generation unit (p)	$2.05\cdot10^{-9}$	Heat and power co-generation unit, 160kW electrical, components for electricity only {GLO}| market for heat and power co-generation unit, 160kW electrical, components for electricity only |
Heat and power co-generation unit (p)	$2.05\cdot10^{-9}$	Heat and power co-generation unit, 160kW electrical, components for heat only {GLO}| market for heat and power co-generation unit, 160kW electrical, components for heat only |
Emissions to air:	**Amount**	**Activity name**
Carbon dioxide, fossil (kg)	$2.30\cdot10^{-2}$	

Carbon monoxide, fossil (kg)	$1.97 \cdot 10^{-5}$
Dinitrogen monoxide (kg)	$1.03 \cdot 10^{-6}$
Methane, fossil (kg)	$9.44 \cdot 10^{-6}$
Nitrogen oxides (kg)	$6.16 \cdot 10^{-6}$
NMVOC, non-methane volatile organic compounds (kg)	$8.21 \cdot 10^{-7}$
Particulates (kg)	$6.16 \cdot 10^{-8}$
Platinum (kg)	$2.87 \cdot 10^{-12}$
Sulfur dioxide vx (kg)	$2.26 \cdot 10^{-7}$

# Pipeline was calculated as in the original activity where $1.43 \cdot 10^{-7}$ km is associated with 1 m^3 of natural gas fossil. Natural gas combustion was needed for the compression as in the original activity.

* To calculate the required kg used in the Sabatier process it was considered the m^3 of natural gas needed in the original activity ($1.05 \cdot 10^{-2}$) and a density of 0.73 kg/m^3.

6. Supplementary results

Table S31. *Distribution of capital cost for the best-case scenario (costs are in a million $) for 100,000 tonnes per year. The following table was made based on the literature data as a guidance.*[4–8]

	S1-BAU	S2-Solar	S3-Wind	S4-OTP	S5-Wind CCS
**Total investment**	168.29	176.48	176.11	168.38	186.26
**Contingency**	8.41	8.91	9.06	8.42	9.56
**Fixed capital investment (FCI)**	176.71	185.39	185.17	176.80	195.83
**Total capital investment (TCI)**	359.82	368.50	368.28	359.91	378.94
**NPV values**	68.50	34.24	29.59	64.58	-8.71

Table S32. *Distribution of capital cost for the worst-case scenario (costs are in million $) for 100,000 tonnes per year. The following table was made based on the literature data as a guidance.*[4–8]

	S1-BAU	S2-Solar	S3-Wind	S4-OTP	S5-Wind CCS
**Total investment**	168.29	207.94	205.67	181.95	235.13
**Contingency**	8.81	10.43	10.54	9.10	12.01
**Fixed capital investment (FCI)**	177.11	218.37	216.21	191.04	247.14
**Total capital investment (TCI)**	312.61	353.87	351.71	326.54	382.64
**NPV values**	38.45	-82.44	-82.09	16.97	-193.24

The LCA Carbon Footprint breakdown activities for each category were given in the following tables:

Table S33. *Carbon footprint results and breakdown in absolute terms per activity footprint for scenarios S1-S4.*

**Scenario (kg CO_2 eq)**	**Total**	**CO_2 embodied**	**EOP**	**H_2O**	**H_2SO_4**	**Protein**	**DAP**	**Enzymes**	**Plant**	**Ethanol from fermentation**	**Grid electricity**	**Type of Cooling**	**Type of Heat**	**Type of Renewable Energy**
S1-BAU	500.51	-77.59	0.48	0.25	0.24	0.93	1.55	39.63	$1.06\cdot10^{-3}$	64.80	18.46	169.88	281.88	
S2-Solar	391.05	-77.59	0.48	0.25	0.24	0.93	1.55	39.63	$1.06\cdot10^{-3}$	64.80	14.77	143.02	201.42	0.86
S3-Wind	261.92	-77.59	0.48	0.25	0.24	0.93	1.55	39.63	$1.06\cdot10^{-3}$	64.80	13.85	101.93	115.29	0.23
S4-OTP	108.31	-77.59	0.32	0.22	0.21	0.56	1.41	39.63	1.06·10-3	51.67	13.34	68.73	17.14	0.55

Table S34. *Carbon footprint results and breakdown in absolute terms per activity for S5-Wind CCS.*

Scenario (kg CO_2 eq)	Total	CO_2 embodied	EOP	H_2O	H_2SO_4	Protein	DAP	Enzyme	Plant	Ethanol from fermentation	Grid electricity	Type of Cooling	Type of Heat	Type of Renewable Energy	CO_2 capture and compression plant	CO_2 storage
S5-Wind CCS	-1.05	-79.57	0.48	0.25	0.24	0.93	1.55	39.63	$1.06.10^{-3}$	64.80	13.85	16.64	-61.90	$7.14\cdot10^{-3}$	1.81	0.01

Table S35. *Carbon footprint results for 1MJ of Heat and breakdown in absolute terms per activity of Heat, district or industrial, Synthetic natural gas from CO_2 DAC and H_2 wind at industrial furnace >100kW.*

Scenario (kg CO_2 eq)	Total	Heat from synthetic natural gas from CO_2 DAC and H2 type	Sabatier process CO_2 DAC type	Natural Gas electricity	Grid electricity	Carbon capture
S5-Wind CCS	$-1.28\cdot10^{-2}$	$7.92\cdot10^{-3}$	$5.11\cdot10^{-5}$	$5.63\cdot10^{-4}$	$8.20\cdot10^{-4}$	$2.50E\cdot10^{-3}$

Table S36. *Carbon footprint results for 1 kg of synthetic natural gas and breakdown in absolute terms per activity of Sabatier process CO_2 DAC Wind.*

**Total (kg CO_2 eq)**	**Synthetic natural gas_Sabatier process onshore wind**	**Grid electricity**
$7.54 \cdot 10^{-3}$	$5.08 \cdot 10^{-3}$	$2.46 \cdot 10^{-3}$

Table S37. *Carbon footprint results for 1 kg of H_2 and breakdown in absolute terms per activity of Hydrogen electrolytic from wind.*

**Total (kg CO_2 eq)**	**Water**	**Potassium hydroxide**	**Onshore Wind electricity**	**Alkaline electrolyzer**	**Electrolyzer production**
$1.40 \cdot 10^{-2}$	$8.34 \cdot 10^{-5}$	$1.08 \cdot 10^{-4}$	$9.77 \cdot 10^{-3}$	$3.49 \cdot 10^{-4}$	$3.69 \cdot 10^{-3}$

Table S38. *Carbon footprint results for 1 kg of CO_2 and breakdown in absolute terms per activity of CO_2 DAC.*

**Total**	**Carbon dioxide DAC**	**Natural gas heat of CO_2 DAC**	**Grid electricity**	**Water**	**Calcium carbonate**
$-5.80 \cdot 10^{-2}$	$-7.70 \cdot 10^{-2}$	$1.03 \cdot 10^{-2}$	$8.02 \cdot 10^{-3}$	$7.51 \cdot 10^{-5}$	$5.67 \cdot 10^{-4}$

Table S39. *Absolute impact results across the scenarios considering the 18 midpoint categories from the ReCiPe 2016 methodology.*

**Impact category**	**Unit**	**S1-BAU**	**S2-SOLAR**	**S3-WIND**	**S4-OTP**	**S5-WIND CCS**
Global warming	kg CO_2 eq	500.51	390.36	261.59	108.32	-1.05
Stratospheric ozone depletion	kg CFC11 eq	$6.54\cdot10^{-4}$	$7.44\cdot10^{-4}$	$6.98\cdot10^{-4}$	$2.12\cdot10^{3}$	$6.96\cdot10^{-4}$
Ionizing radiation	kBq Co-60 eq	32.17	72.59	61.89	36.28	62.42
Ozone formation, Human health	kg NOx eq	0.95	1.25	0.91	342.18	11.27
Fine particulate matter formation	kg PM2.5 eq	0.58	0.91	0.65	28.29	1.49
Ozone formation, Terrestrial ecosystems	kg NOx eq	1.02	1.31	0.95	987.34	30,91
Terrestrial acidification	kg SO_2 eq	1.80	2.46	1.90	659.87	5.41
Freshwater eutrophication	kg P eq	0.12	0.20	0.13	0.20	0.13
Marine eutrophication	kg N eq	0.14	0.15	0.14	0.31	0.14
Terrestrial ecotoxicity	kg 1,4-DCB	1817.23	6436.24	3070.25	1804.03	2953.19
Freshwater ecotoxicity	kg 1,4-DCB	22.07	64.48	40.24	23.69	47.09
Marine ecotoxicity	kg 1,4-DCB	24.96	80.23	47.82	87324.91	55.87

Human carcinogenic toxicity	kg 1,4-DCB	20.61	56.82	58.48	1248.96	60.92
Human non-carcinogenic toxicity	kg 1,4-DCB	447.04	922.99	616.85	73106.13	592.60
Land use	m^2a crop eq	119.76	178.39	119.40	443.75	121.72
Mineral resource scarcity	kg Cu eq	1.51	5.02	4.33	1.77	4.29
Fossil resource scarcity	kg oil eq	163.36	110.16	77.53	48.25	77.28
Water consumption	m^3	19.25	27.06	21.83	31.82	43.20

7. References

(1) Servian-Rivas, L. D.; Pachón, E. R.; Rodríguez, M.; González-Miquel, M.; González, E. J.; Díaz, I. Techno-Economic and Environmental Impact Assessment of an Olive Tree Pruning Waste Multiproduct Biorefinery. *Food and Bioproducts Processing*. **2022**, *134*, 95–108. DOI 10.1016/j.fbp.2022.05.003.

(2) Gómez-Cruz, I.; Contreras, M.d.M; Romero, I.; Castro, E. A Biorefinery Approach to Obtain Antioxidants, Lignin and Sugars from Exhausted Olive Pomace. *Journal of Industrial and Engineering Chemistry*. **2021**, 96, 356–363. DOI 10.1016/j.jiec.2021.01.042.

(3) Turton; Richard; Bailie; Richard C; Whiting; Wallace B; Shaeiwitz; Joseph A. Analysis, Synthesis, and Design of Chemical; 5TH ed; Pearson, **2018**.

(4) Brown, T. R. A Critical Analysis of Thermochemical Cellulosic Biorefinery Capital Cost Estimates. *Biofuels, Bioproducts and Biorefining*. **2015**, *9* (4), 412–421. DOI 10.1002/BBB.1546.

(5) Makepa, D. C.; Chihobo, C. H.; Ruziwa, W. R.; Musademba, D. A Systematic Review of the Techno-Economic Assessment and Biomass Supply Chain Uncertainties of Biofuels Production from Fast Pyrolysis of Lignocellulosic Biomass. *Fuel Communications*. **2023**, *14*, 100086. DOI 10.1016/J.JFUECO.2023.100086.

(6) Figueroa-Torres, G. M.; Theodoropoulos, C. Techno-Economic Analysis of a Microalgae-Based Biorefinery Network for Biofuels and Value-Added Products. *Bioresour Technol Rep*. **2023**, *23*, 101524. DOI 10.1016/J.BITEB.2023.101524.

(7) Borello, D.; De Caprariis, B.; De Filippis, P.; Carlo, A. Di; Marchegiani, A.; Pantaleo, A. M.; Shah, N.; Venturini, P. Thermo-Economic Assessment of an Olive Pomace Gasifier for Cogeneration Applications Selection and/or Peer-Review under Responsibility of ICAE. *Energy Procedia*. **2015**, *75*, 252–258. DOI 10.1016/j.egypro.2015.07.325.

(8) Towler, G.; Sinnott, R.; Boston, A. •; Heidelberg, •; London, •; San, P. •; San, D.; Singapore, F. •; Tokyo, S. •. *CHEMICAL ENGINEERING DESIGN Principles, Practice and Economics of Plant and Process Design*; **2008**.

(9) Ramos, J. S.; Ferreira, A. F. Techno-Economic Analysis and Life Cycle Assessment of Olive and Wine Industry Co-Products Valorisation. *Renewable and Sustainable Energy Reviews*. **2022**, *155*.111929 DOI 10.1016/j.rser.2021.111929.

(10) Mesa, L.; López, N.; Cara, C.; Castro, E.; González, E.; Mussatto, S. I. Techno-Economic Evaluation of Strategies Based on Two Steps Organosolv Pretreatment and Enzymatic Hydrolysis of Sugarcane Bagasse for Ethanol Production. *Renew Energy*. **2015**, 86, 270–279. DOI 10.1016/j.renene.2015.07.105

(11) Susmozas, A.; Moreno, A. D.; Romero-García, J. M.; Manzanares, P.; Ballesteros, M. Designing an Olive Tree Pruning Biorefinery for the Production of Bioethanol, Xylitol and Antioxidants: A Techno-Economic Assessment. *Holzforschung*. **2019**, *73* (1), 15–23. DOI 10.1515/hf-2018-0099.

(12) Guilera, J.; Ramon Morante, J.; Andreu, T. Economic Viability of SNG Production from Power and CO_2. *Energy Convers Manag*. **2018**, *162*, 218–224. DOI 10.1016/J.ENCONMAN.2018.02.037.

(13) Laude, A.; Ricci, O.; Bureau, G.; Royer-Adnot, J.; Fabbri, A. CO_2 Capture and Storage from a Bioethanol Plant: Carbon and Energy Footprint and Economic Assessment. *International Journal of Greenhouse Gas Control*. **2011**, *5* (5), 1220–1231. DOI 10.1016/J.IJGGC.2011.06.004.

(14) *Levelised cost of CO_2 capture by sector and initial CO_2 concentration, 2019 – Charts – Data & Statistics - IEA*. https://www.iea.org/data-and-statistics/charts/levelised-cost-of-CO_2-capture-by-sector-and-initial-CO_2-concentration-2019 (accessed 2024-01-22).

(15) Szima, S.; Cormos, C. C. CO_2 Utilization Technologies: A Techno-Economic Analysis for Synthetic Natural Gas Production. *Energies (Basel)*. **2021**, *14* (5), 1258. DOI 10.3390/en14051258.

(16) Guilera, J.; Ramon Morante, J.; Andreu, T. Economic Viability of SNG Production from Power and CO_2. *Energy Convers Manag*. **2018**, *162*, 218–224. DOI 10.1016/J.ENCONMAN.2018.02.037.

(17) Fasihi, M.; Efimova, O.; Breyer, C. Techno-Economic Assessment of CO_2 Direct Air Capture Plants. *J Clean Prod*. **2019**, *224*, 957–980. DOI 10.1016/j.jclepro.2019.03.086.

(18) Medrano-García, J. D.; Charalambous, M. A.; Guillén-Gosálbez, G. Economic and Environmental Barriers of CO_2-Based Fischer-Tropsch Electro-Diesel. *ACS Sustain Chem Eng*. **2022**, *10* (36), 11751–11759. DOI 10.1021/acssuschemeng.2c01983.

(19) International Renewable Energy Agency. *RENEWABLE POWER GENERATION COSTS IN 2018*; Abu Dhabi, **2018.**

(20) *Levelized cost of heating (LCOH) for consumers, for selected space and water heating technologies and countries – Charts – Data & Statistics - IEA*. https://www.iea.org/data-and-statistics/charts/levelized-cost-of-heating-lcoh-for-consumers-for-selected-space-and-water-heating-technologies-and-countries (accessed 2024-01-22).

(21) European Commission: Directorate-General for Energy, Heald, S., Debrosses, N., Rademaekers, K., Moerenhout, J., Altman, M., Yearwood, J., Pollier, K., Smith, M., Saheb, Y., Badouard, T., Pollitt, H., & Peffen, A. (2018). Study on energy prices, costs and subsidies and their impact on industry and households: final report, *Publications Office.* **2018**. DOI 10.2833/825966.

(22) Badouard, T.; Moreira De Oliveira, D.; Yearwood, J.; Torres, P. Rotterdam, 31 July 2020 Client: European Commission-DG Energy A4 Final Report-Cost of Energy (LCOE) Study on Energy Costs, Taxes and the Impact of Government Interventions on Investments in the Energy Sector. **2020**.

(23) Volkart, K.; Bauer, C.; Boulet, C. Life Cycle Assessment of Carbon Capture and Storage in Power Generation and Industry in Europe. *International Journal of Greenhouse Gas Control* **2013**, *16*, 91–106. DOI 10.1016/J.IJGGC.2013.03.003.

(24) Bello, S.; Galán-Martín, Á.; Feijoo, G.; Moreira, M. T.; Guillén-Gosálbez, G. BECCS Based on Bioethanol from Wood Residues: Potential towards a Carbon-Negative Transport and Side-Effects. *Appl Energy*. **2020**, *279*, 115884. DOI 10.1016/J.APENERGY.2020.115884.

(25) Gerloff, N. Comparative Life-Cycle-Assessment Analysis of Three Major Water Electrolysis Technologies While Applying Various Energy Scenarios for a Greener Hydrogen Production. *J Energy Storage*. **2021**, *43*, 102759. DOI 10.1016/J.EST.2021.102759.

(26) Bargiacchi, E.; Candelaresi, D.; Valente, A.; Spazzafumo, G.; Frigo, S. Life Cycle Assessment of Substitute Natural Gas Production from Biomass and Electrolytic Hydrogen. *Int J Hydrogen Energy*. **2021**, *46* (72), 35974–35984. DOI 10.1016/j.ijhydene.2021.01.033.

(27) Fernández-Lobato, L., López-Sánchez, Y., Blejman, G., Jurado, F., Moyano-Fuentes, J., & Vera, D. Life Cycle Assessment of the Spanish Virgin Olive Oil Production: A Case Study for Andalusian Region. *Journal of Cleaner Production*. **2020**, 290, 125677. DOI 10.1016/j.jclepro.2020.125677.

(28) Candelaresi, D.; Valente, A.; Iribarren, D.; Dufour, J.; Spazzafumo, G. Comparative Life Cycle Assessment of Hydrogen-Fuelled Passenger Cars. *Int J Hydrogen Energy*. **2021**, *46* (72), 35961–35973. DOI 10.1016/j.ijhydene.2021.01.034.

(29) Proto, A. R.; Palma, A.; Paris, E.; Papandrea, S. F.; Vincenti, B.; Carnevale, M.; Guerriero, E.; Bonofiglio, R.; Gallucci, F. Assessment of Wood Chip Combustion and Emission Behavior of Different Agricultural Biomasses. *Fuel*. **2021**, *289*, 119758. DOI 10.1016/j.fuel.2020.119758.

(30) AgroBioHeat_factsheet_olive_stones_en. **2020**.
